# Genistein induces long-term expression of progesterone receptor regardless of estrogen receptor status and improves the prognosis of endometrial cancer patients

**DOI:** 10.1038/s41598-022-13842-6

**Published:** 2022-06-18

**Authors:** Kaori Yoriki, Taisuke Mori, Kohei Aoyama, Yosuke Tarumi, Hisashi Kataoka, Tetsuya Kokabu, Jo Kitawaki

**Affiliations:** Department of Obstetrics and Gynecology, Graduate School of Medical Science, Kyoto Prefectural University of Medicine, 465 Kajii-cho, Kawaramachi-Hirokoji, Kamigyo-ku, Kyoto, 602-8566 Japan

**Keywords:** Endometrial cancer, Cancer therapeutic resistance, Hormone receptors

## Abstract

Progesterone is used to treat uterine endometrial cancer in young patients wishing to preserve their fertility as well as in advanced or recurrent patients, but its response rate is limited. The antitumor effect of progesterone is mediated by progesterone receptor (PR) binding. Hence, loss of progesterone’s therapeutic effect, i.e., development of progesterone resistance, is mainly due to decreased PR expression. However, little is known about underlying mechanisms that regulate PR expression. Immunohistochemistry analysis of specimens from 31 young, endometrial cancer patients showed that elevated PR expression significantly increased (*P* < 0.05) rates of progression-free and overall survival. We investigated mechanisms of regulating PR expression and suppressing cell proliferation using genistein, a chemotherapeutic agent against different cancers. Genistein inhibits cell growth by inducing cell cycle arrest in G2 and apoptosis; moreover, it upregulates prolonged expression of PR-B and forkhead box protein O1, regardless of estrogen receptor alpha expression in endometrial cancer cells. Genistein-induced PR expression decreases CCAAT/enhancer binding protein beta expression and activates c-Jun N-terminal kinase pathway, rather than causing epigenetic alterations of the PR promoter. Therefore, increased PR expression is an important antitumor effect of genistein. This may help to improve the response rates of fertility-sparing treatments for young patients.

## Introduction

Uterine endometrial cancer is a common gynecological malignancy of the female urogenital tract, and its incidence and death rates have been notably increasing^1^. From 1990 to 2017, the age-standardized incidence and prevalence rate increased globally by 0.58 and 0.89% per year, respectively^2^. The main characteristic feature of endometrial cancer is its estrogen-dependent tumorigenesis. Prolonged exposure to unopposed estrogen can cause endometrial hyperplasia and cancer in a normal endometrium^3^. Since progesterone causes atrophy of the normal endometrium, high-dose progestin is administered clinically as a single agent in the treatment of endometrial cancer^4^, and it has been demonstrated to be effective in many clinical trials. In fact, one review reported that its response rates for atypical endometrial hyperplasia and well-differentiated endometrioid carcinoma localized to the endometrium were 86% and 75%, respectively^5^. Progesterone effects are mostly mediated via the progesterone receptor (PR) and are associated with the levels of PR activity. However, the molecular mechanisms regulating PR expression and the anti-tumor mechanisms of PR signaling remain unknown. Therefore, the clinical use of progesterone is limited due to the lack of biomarkers that can predict the hormone sensitivity.

The human *PR* gene encodes two proteins, namely the 120 kDa PR-B and the 94 kDa PR-A. Incidentally, PR-B is the full-length form that has an additional 164 amino acids at the N-terminus including an additional transactivation function (AF3) region^6^. Several reports have shown that the functional activities of PR-A and PR-B differ in a cell type-, promoter-, or ligand-specific manner^7–9^. Hence, it is possible that the different mechanisms for regulating the PR isoforms are reflected through the differential expression of the PRs in the endometrium. It has been hypothesized that two promoters, namely Promoter A (+ 464 to + 1105) and Promoter B (− 711 to + 31), are responsible for the production of PR-A and PR-B, respectively. Interestingly, neither PR-A nor PR-B contains a palindromic estrogen response element (ERE). A previous report had proposed that the estrogen responsiveness of the human *PR* gene is derived in part from the interaction of estrogen receptor α (ERα) and specificity protein 1 (Sp1) with a region in Promoter A lying between + 571 and + 595; this site contains a half ERE site and two adjacent Sp1 sites, and it is referred to as the + 571 ERE/Sp1 site^10^. In contrast, a large proportion of in vitro evidence suggests that the absence or reduced expression of PR-B, but not PR-A, might result in the failure of progesterone treatment; this is called progesterone resistance, and it leads to aberrant PR-B-mediated signaling in endometrial cancer cells^11^. More than 30% of patients with estrogen-dependent and well-differentiated endometrial cancer fail to respond to progesterone treatment due to this progesterone resistance^12^. Hence, to overcome this, it is essential to clarify the underlying mechanisms of progesterone resistance.

Genistein is a biologically active isoflavone found in soy products. It has been shown to modulate several pathophysiological pathways that are commonly deregulated in obesity, metabolic syndromes, and cancer. The structural similarities between genistein and estradiol can also elucidate its potential role in treating postmenopausal symptoms, such as reduction in bone mass and hot flushes^13^. Genistein also has an anti-proliferative effect on various cancer cells, including endometrial cancer^14^. There are a few reports on the action of genistein on hormone receptors in endometrial cancer cells; however, one study has indicated that genistein decreases ERα mRNA expression while increasing *PR* expression^15^. Therefore, we examined the genistein-induced expression of each PR isoform and clarified its role in PR regulation as well as its PR-mediated antitumor effects.

## Results

### Expression of PR in endometrial cancer is linked to prognosis in young patients

**Figure 1:**
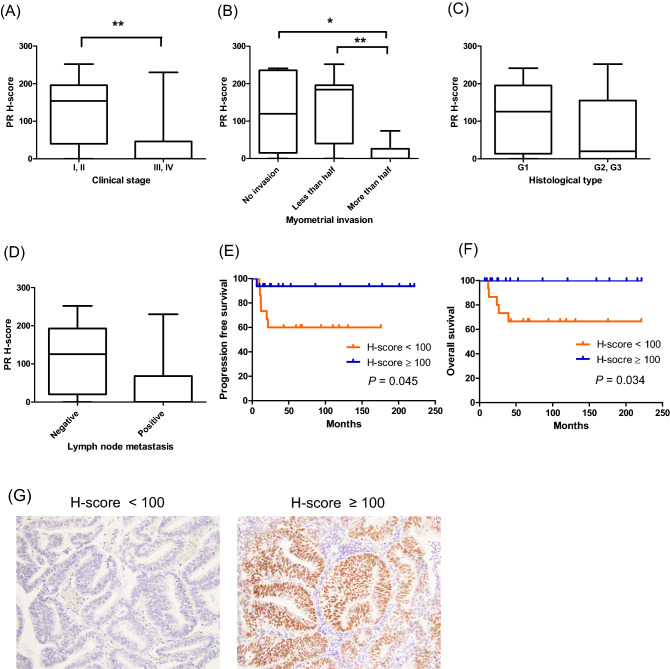
Expression of progesterone receptor (PR) in endometrial tissues of young patients with endometrial cancer. (**A**)–(**C**) Association of PR expression with clinico-pathological factors. The PR expression levels examined by immunohistochemical analysis were evaluated by H-score, and their association with clinical parameters, including clinical stage based on FIGO 2008 classification (**A**), myometrial invasion (**B**), histopathological type (**C**), and lymph node metastasis (**D**) are presented. Statistical significance was determined by using two-tailed unpaired Student’s t-test and one-way analysis of variance (ANOVA) with post-hoc Tukey’s multiple comparison test. Kaplan–Meier analysis of progression-free survival (**E**) and overall survival (**F**) is shown in association with PR expression (**G**). *P < 0.05; **P < 0.01.

First, we performed immunohistochemical analysis of tissue specimens collected from endometrial cancer patients aged < 40 years. Patient characteristics are shown in Table 1. Of the 32 patients’ specimens, 31 were available for analysis. The H-score distribution of PR is presented in Table 2. Incidentally, the H-score of PR is significantly associated with the clinical stage (between FIGO stage I or II and III or IV, *P* < 0.01) of the disease. Moreover, it is significantly related to myometrial invasion (*P* < 0.05 between cases with no invasion and those with > 50% myometrial invasion, and *P* < 0.01 between cases with < 50% myometrial invasion and those with > 50% myometrial invasion) (Fig. 1A, B). However, there are no significant associations between PR expression and the histological type or lymph node metastasis (Fig. 1C, D). The Kaplan–Meier analysis indicated that the level of PR expression is correlated with progression-free survival (*P* < 0.05, Fig. 1E) and overall survival (*P* < 0.05, Fig. 1F) when the cases were divided into two groups by PR-H-score 100 (Fig. 1G), thereby suggesting that PR expression could be an independent prognostic factor for endometrial cancer.

**Table 1:** Patient characteristics.

	N = 31
Mean age (range)	36.2 (27–39)
Histological type	
Endometrioid	31 (100%)
Others	0 (0%)
Histological grade	
1	23 (74.2%)
2	7 (22.6%)
3	1 (3.2%)
Myometrial invasion	
No invasion	6 (19.4%)
Less than half	16 (51.6%)
More than half	9 (29.0%)
Lymph node metastasis	
Negative	25 (80.6%)
Positive	6 (19.4%)
Stage	
I	16 (51.6%)
II	5 (16.1%)
III	6 (19.4%)
IV	4 (12.9%)
Total	31 (100%)

**Table 2:** Distribution of PR-H-score.

H-score	Patient number (%)
1–50	14 (45.2%)
51–100	1 (3.2%)
101–200	10 (32.2%)
201–300	6 (19.4%)
Total	31 (100%)

### Genistein inhibits cell growth by inducing cell cycle arrest in G2 and apoptosis in endometrial cancer cells

**Figure 2:**
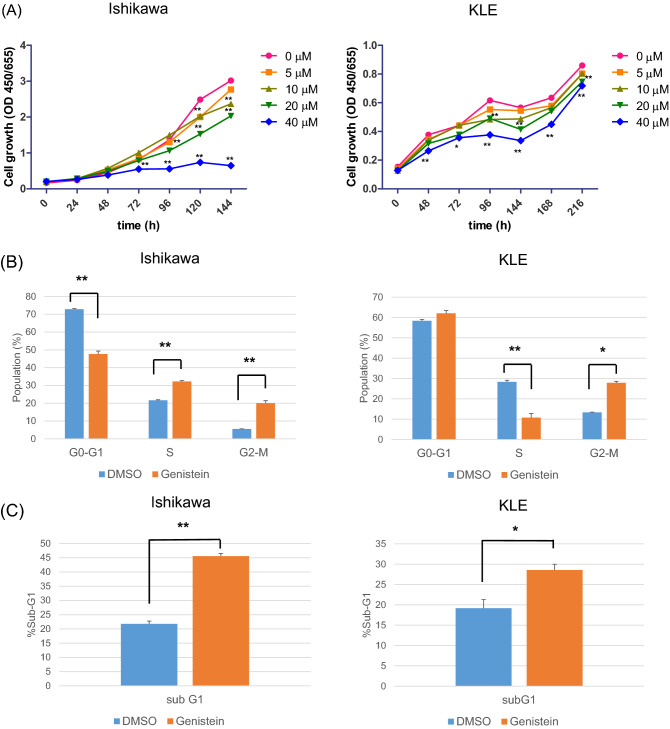

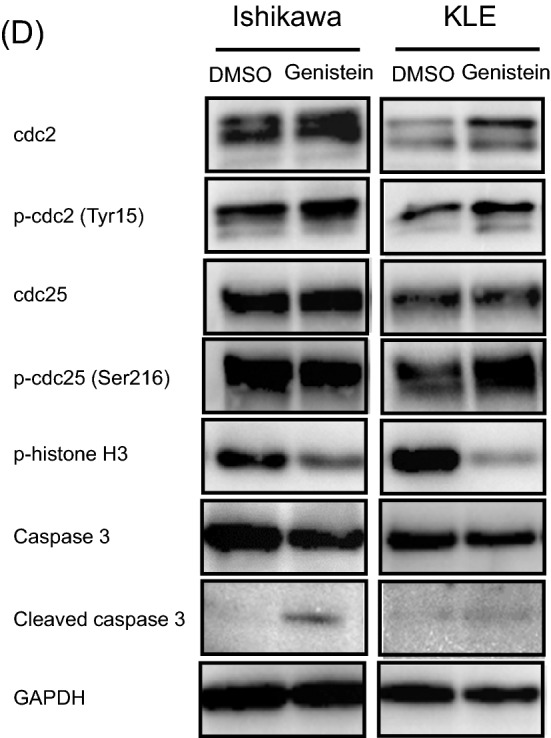
Effects of genistein on the proliferation of cancer cells. The water-soluble tetrazolium salt-8 (WST-8) cell proliferation assays were performed for Ishikawa and KLE cells (**A**). The viability of cancer cells was determined every 24–48 h post-treatment with 20 μM genistein. Data are represented as the mean value (n = 12). Statistical significance was determined using a two-way analysis of variance (ANOVA). Cell cycle analysis was performed using flow cytometry. Cancer cells were collected, and flow cytometry analysis was performed 144 h after exposure to genistein. Cell distribution at each phase of the cell cycle (**B**). Data represent the mean ± standard error of the mean (n = 3). Sub-G1 population of both cells (**C**). Data represent the mean ± standard error of the mean (n = 3). Western blot analysis of proteins involved in the G2/M phase (phospho-histone H3 (Ser10), cdc2, phospho-cdc2 (Tyr15), cdc25, and phosho-cdc25 (Ser216)) and apoptosis (caspase 3 and cleaved caspase 3) after 48 h of genistein treatment (**D**). Full-length western blots are presented in Supplementary Fig. 3. *P* values are based on Student’s *t*-test. *P < 0.05; **P < 0.01.

To examine the effect of genistein on cell proliferation in endometrial cancer cell lines, namely ERα-dependent Ishikawa and ERα-independent KLE, we performed the water-soluble tetrazolium salt-8 (WST-8) assay. Interestingly, genistein significantly inhibits cell growth in a dose- and time-dependent manner in both the cell lines (Fig. 2A). Subsequently, in order to evaluate the underlying mechanism of growth inhibition by genistein, we performed flow cytometry analysis. Ishikawa and KLE cells were treated with 20 μM genistein for 6 days, and it caused the accumulation of cells in the G2/M- and sub-G1-phases (Fig. 2B, C). Our western blot analysis also showed a decrease in phospho-histone H3 (Ser10), a representative marker of mitotic phase, and an increase in cdc2 phosphorylation after 48 h of exposure to genistein in both the cells; however, there was no change in cdc25 phosphorylation (Fig. 2D). We also detected an increase in cleaved caspase-3, thereby indicating the initiation of apoptosis. These results indicate that genistein induces cell cycle arrest at the G2/M phase in endometrial cancer cells, followed by their apoptosis.

### Genistein upregulates the expression of PR and forkhead box protein O1 (FOXO1) regardless of ERα expression in endometrial cancer cells

Next, we performed quantitative polymerase chain reaction (qPCR) assays and western blot analyses to elucidate the effect of genistein on PR expression in endometrial cancer cell lines. For that, we first confirmed that Ishikawa cells show higher expression of ERα and lower expression of PR-B, as compared to that in KLE cells (Supplementary Fig. 1). There is no significant difference in ERß expression between the two cells, and PR-A expression is low in both the cell lines.

**Figure 3:**
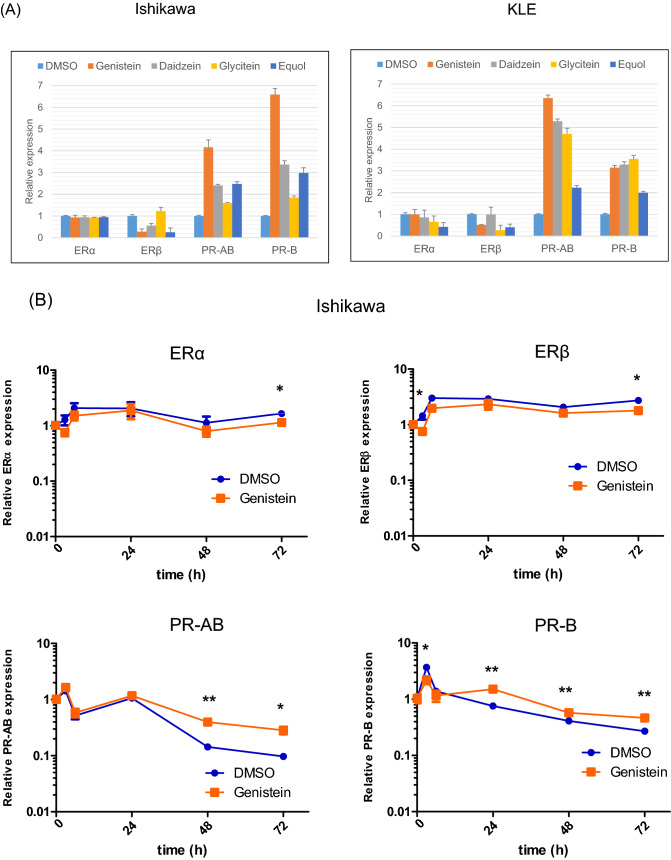

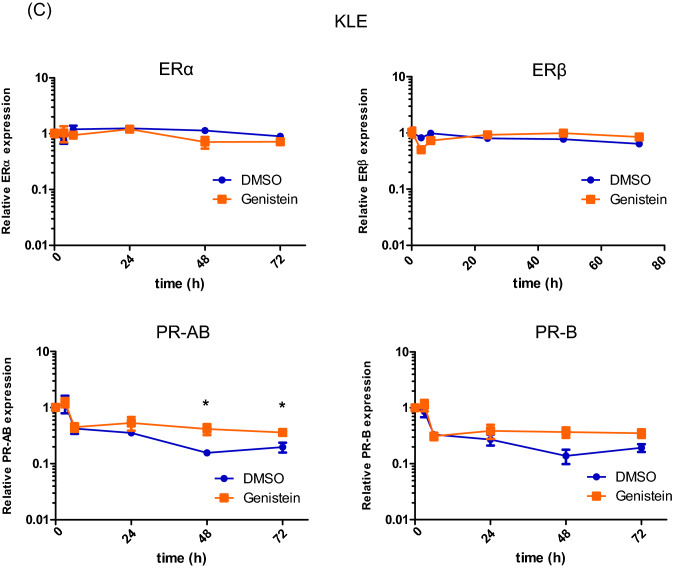

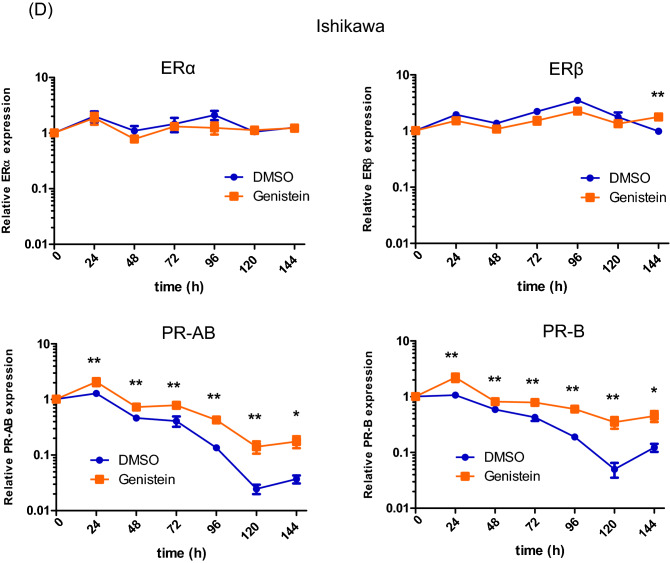

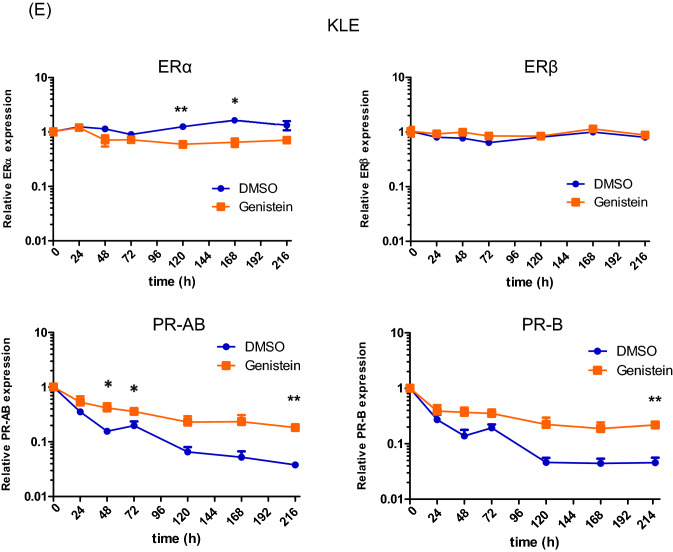

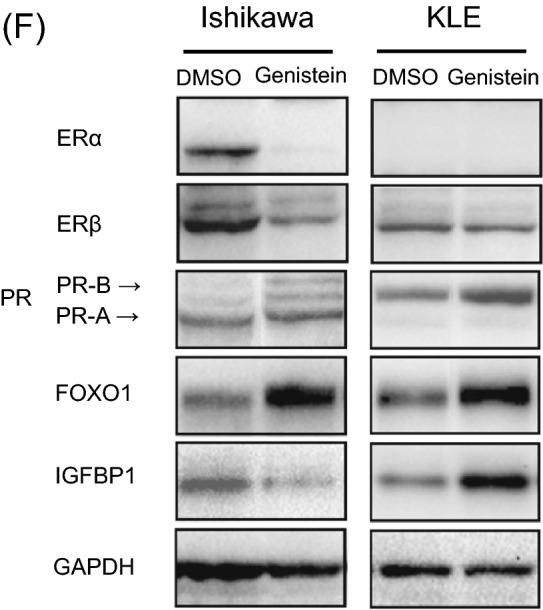
Effects of genistein on expression of estrogen receptor (ER)α, ERβ, progesterone receptor (PR)-AB, and PR-B. The expressions of ERα, ERβ, PR-AB, and PR-B were examined by quantitative polymerase chain reaction (qPCR) analysis in Ishikawa and KLE cells after exposure to 20 μM genistein, daidzein, glycitein, and equol (**A**). The expression of the hormone receptors in Ishikawa (**B**) and KLE cells (**C**) was measured using qPCR after 0, 3, 6, 24, 48, and 72 h of 20 μM genistein treatment without medium replacement. The receptor expressions were measured every 2 or 3 d for 6 d in Ishikawa cells with medium replacement every day (**D**) and measured every 1 or 2 d for 9 d in KLE cells (**E**). The relative expression values are displayed in logarithms with a base of 2. Data represent the mean ± standard error of the mean (n = 3). Western blot analysis of these hormone receptors, forkhead box protein O1 (FOXO1), and insulin-like growth factor-binding protein 1 (IGFBP1) in cancer cells treated with genistein for 144 h (**F**). Full-length western blots are presented in Supplementary Fig. 4. Data represent the mean ± standard error of the mean (n = 3). *P* values are based on Student’s *t*-test. *P < 0.05; **P < 0.01.

We evaluated the differences in ER and PR expression owing to the effect of the specific isoflavone treatment, namely genistein, daidzein, glycitein, and equol. The expression of ERα, ERß, PR-AB, and PR-B was examined by qPCR in Ishikawa and KLE cells treated with each of the isoflavone metabolites (20 μM) for 6 days. We observed that genistein upregulates PR-AB expression to the highest level, as compared to the effect of other isoflavone metabolites, without increasing the expressions of ERα and ERß in both the cancer cell lines (Fig. 3A).

To elucidate the effect of genistein on ER and PR expressions over a short period without medium replacement, both the receptor expressions were assessed after 0, 3, 6, 24, 48 and 72 h of treatment with 20 μM genistein. We observed that genistein significantly induces the expression of PR-B in Ishikawa cells and that of PR-AB in Ishikawa and KLE cells after 48 h, but it does not change the expressions of ERα and ERß in either of the cell lines (Fig. 3B, C). Similarly, the effect of genistein over a long period with replacement of the medium every 2 or 3 days was examined every day for 6 days in the Ishikawa cells and every 1 or 2 days for 9 days in the KLE cells. We observed that the effects of genistein on increasing PR-AB and PR-B expressions are retained until the final time point in both the cell lines (Fig. 3D, E). To further confirm this observation, we used western blot analyses to assess the expressions of ER, PR, FOXO1, a tumor suppressor located downstream of PR signaling, and insulin-like growth factor-binding protein 1 (IGFBP1), a factor associated with the decidua. Interestingly, genistein stimulates the expression of PR-B and FOXO1 in Ishikawa and KLE cells (Fig. 3F). Unlike the qPCR results, genistein reduces the expression of ERα and ERß in Ishikawa cells. Moreover, IGFBP1 expression is increased in KLE cells, although its expression is reduced in Ishikawa cells.

### Genistein downregulates CCAAT/enhancer binding protein beta (C/EBPß) and activates c-Jun N-terminal kinase (JNK) pathway

**Figure 4:**
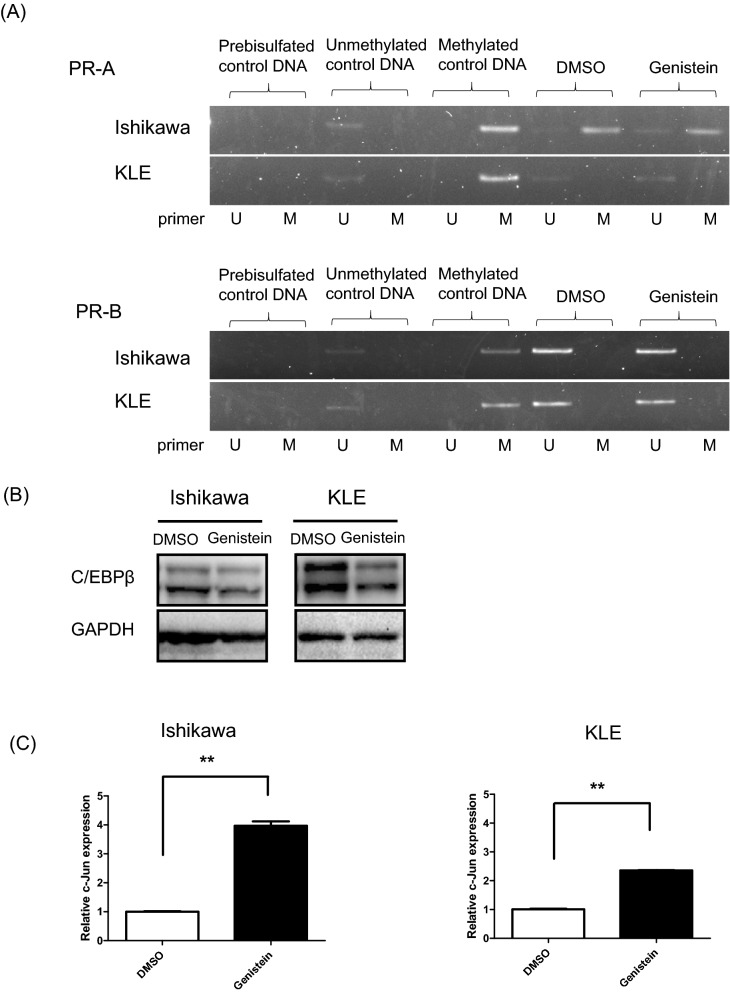

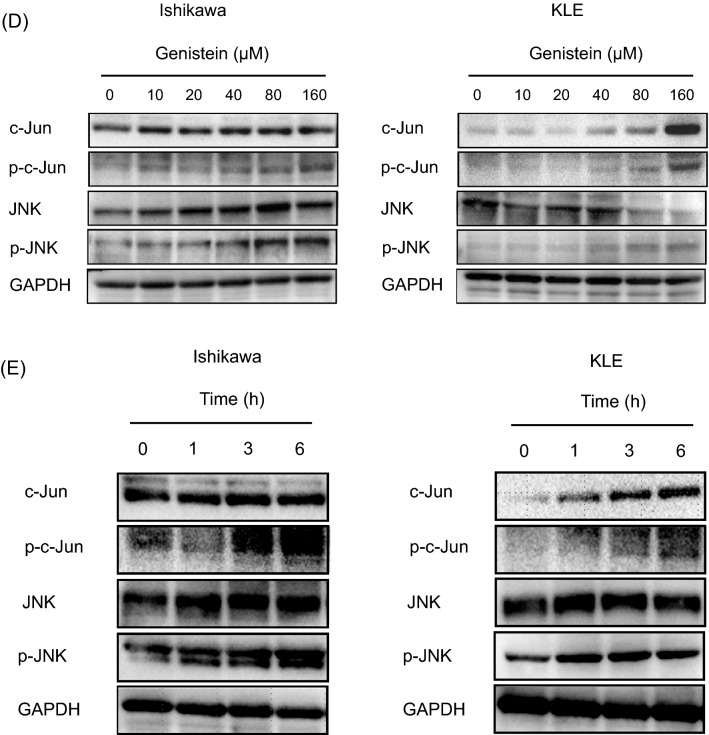
Effects of genistein on the methylation of progesterone receptor (PR) promoter, the expression of CCAAT/enhancer binding protein beta (C/EBPβ), and the activation of c-Jun N-terminal kinase (JNK) pathway. The methylation status of promoters of PR-A and PR-B was assessed in Ishikawa and KLE cells treated with genistein for 144 h using methylation-specific polymerase chain reaction (MSP) (**A**). The expression of C/EBPβ in cancer cells was evaluated by western blot analysis (**B**). The expression of c-Jun in cancer cells was examined by quantitative polymerase chain reaction (qPCR) (**C**). Western blot analysis of proteins related to the JNK pathway in cancer cells after 24 h of exposure to 0, 10, 20, 40, 80, and 160 μM genistein (**D**) and at 0, 1, 3, and 6 h after treatment with 100 μM genistein (**E**). Full-length western blots are presented in Supplementary Fig. 5–6. Data represent the mean ± standard error of the mean (n = 3). *P* values are based on Student’s *t*-test. **P < 0.01.

We hypothesized that PR promoter unmethylation leads to the genistein-induced PR expression in endometrial cancer. The influence of genistein on the methylation of the PR promoter was assessed in endometrial cancer cell lines using methylation-specific PCR (MSP). We observed that the Ishikawa and KLE cells have little CpG methylation on the PR-B promoter (Fig. 4A). Hence, treatment with genistein does not induce epigenetic alterations of the PR-B promoter in either of the cell lines, and genistein is not involved in the demethylation of the PR-B promoter. Next, we selected the candidates for transcription factors with sequences predicted to bind to the PR-B promoter using JASPAR. Furthermore, the expressions of C/EBPβ and c-Jun, which have been reported as transcription factors in previous papers^16,17^, were investigated. Incidentally, C/EBPβ suppresses PR expression, whereas c-Jun stimulates PR expression. Genistein reduces the expression of C/EBPβ in Ishikawa and KLE cells (Fig. 4B). Moreover, in addition to the increase of c-Jun mRNA levels, genistein treatment leads to the increased protein expression of c-Jun and phosphorylation of JNK in a dose- and time-dependent manner (Fig. 4C–E).

### Effect of genistein on tumor growth in a mouse xenograft model

**Figure 5:**
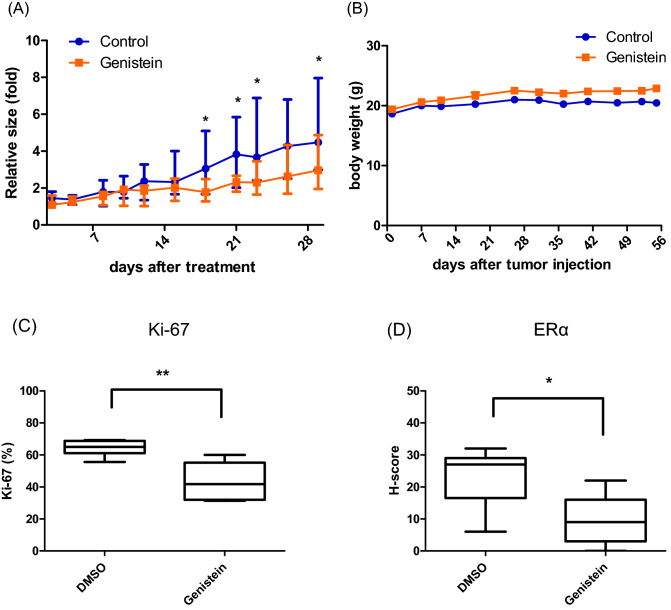
Effects of genistein on in vivo endometrial cancer cell growth using a mouse xenograft model. To analyze in vivo tumor growth, xenograft model mice inoculated with Ishikawa cells were treated by intraperitoneal injection of vehicle or genistein (90 mg/kg body weight) on alternate days for four weeks. Tumor volume in genistein and control groups during experiments (complete data shown in Supplementary Table 1) (**A**). Proliferation rate calculated on the basis of Ki-67-immunopositive cells in tumor sections (**C**). Body weight of the genistein and control groups (**B**). Estrogen receptor alpha (ERα) levels were immunohistochemically evaluated by H-score (**D**). Data represent the mean ± standard error of the mean (n = 6). *P* values are based on Student’s *t*-test. *P < 0.05; **P < 0.01.

To further evaluate the effect of genistein on endometrial cancer cells in vivo, athymic nude mice were subcutaneously inoculated with Ishikawa cells. We observed that genistein significantly suppresses tumor growth, as compared to the control group, without reducing body weight (Fig. 5A, B). Thereafter, we analyzed post-treatment apoptosis in the tumors using the TUNEL assay. There was no difference in apoptosis between the genistein-treated and control groups (Supplementary Fig. 8). The tissue sections were also immunohistochemically stained with Ki-67, a representative cell proliferation marker. Genistein treatment significantly reduces the percentage of Ki-67-positive cells (Fig. 5C). Additionally, we assessed the effect of genistein on ERα and FOXO1 expressions in vivo using immunostaining. Genistein treatment significantly reduces the expression of ERα in the tumors (Fig. 5D).

## Discussion

We revealed that PR expression is associated with favorable clinical outcomes in young patients with endometrial cancer who wish to preserve their fertility. Moreover, we elucidated the ER-independent inhibition of cell proliferation as well as the mechanism of increased PR expression in endometrial cancer using genistein. In previous studies using qPCR and immunostaining, there have been various reports on PR isoform status related to prognosis, namely PR-A and/or PR-B^18–21^. Therefore, it is natural that an absence of the PR status is associated with poor prognosis. Our study obtained similar results, even though it was limited to endometrial cancer in young patients aged < 40 years, thereby suggesting that increased PR expression leads to improved survival, regardless of progestin therapy. These findings are important for the development of treatment options for patients with endometrial cancer who desire to preserve their fertility.

First, our results demonstrated that genistein suppresses cell proliferation by inducing cell cycle arrest at the G2-M phase as well as apoptosis, and it upregulates the expression of FOXO1. Although our study did not find a convincing apoptotic effect of genistein via the TUNEL assay, a previous study has reported that it increased the expression of Bax, Bad, and Bak in vivo which promoted tumor apoptosis^22^. The discrepancy in these findings may be related to the use of different analytical methods for the detection of apoptosis. Many previous studies have reported that genistein causes G2/M block in cell cycle progression and suppresses cell proliferation in vitro and in vivo not only in endometrial cancer, but also in cases of breast cancer, prostate cancer, hepatic cancer, and bladder cancer^15,22–28^. However, the concentration of genistein has opposite effects on cell growth in breast cancer^29^. Particularly, the anti-proliferative effects of genistein that are mainly observed at high treatment doses (> 20 μM) are independent of ER, while the proliferative effects observed at low treatment doses (0.01–10 μM) are ER-mediated^30–34^. In ER-negative cells, this dual effect is not observed, and genistein produces only anti-proliferative effects, especially at high doses^35,36^. Similarly, in our study, ER-positive Ishikawa cells exhibited a low cell growth after 72 and 96 h of genistein exposure, while ER-negative KLE cells did not show this change. The FOXO family, including FOXO1, is a key effector of phosphoinositide 3-kinase (PI3K) deregulation since it is a direct downstream phosphorylation target of the protein kinase PKB and the related kinase SGK1^37,38^. About 30–50% of endometrioid carcinomas have mutations in the *PI3KCA* gene, which enhances the activity of the PI3K/PKB signaling pathway^39^. Incidentally, FOXO1 is upregulated by progestin in a PR-B-dependent manner, and it is involved in cell cycle inhibition, apoptosis, and inhibition of migratory and invasive capacities in endometrial cancer^40–43^. Although the crosstalk between FOXO1 and PR is not strictly ligand-dependent, this crosstalk is important for decidualization as well as the induction of apoptosis^44,45^. Additionally, genistein has been reported to decrease the phosphorylation of Akt and increase the phosphorylation of p42/44 (ERK) in endometrial cancer cell lines^15^. Our data suggest that genistein inhibits cell proliferation by inducing the expression of FOXO1, which lies in the downstream of PI3K/PKB signaling pathway, regardless of ER status in endometrial cancer.

Second, our results showed that genistein significantly increases the expressions of PR-AB and PR-B after 24 h of treatment, regardless of the ER status of the cell lines, and the effect was sustained up to 144 h in Ishikawa cells and 216 h in KLE cells. In fact, the C/EBPβ and the JNK pathway participated in the genistein-induced elevation of PR expression, rather than the estrogen responsiveness or epigenetic modification. One of the mechanisms for regulating the *PR* gene is by mediating estrogen responsiveness, even though the *PR* gene lacks a palindromic ERE sequence^6^. The promoter of PR-A contains a half ERE site upstream of two adjacent Sp1 sites, and the ER and Sp1 may play a role in activating the PR-A promoter^10,46^. However, our study suggests that the genistein-stimulated PR expression is not ER-mediated because genistein hardly changed the mRNA levels of ERα and ERβ; moreover, it decreased the protein level of ERα in the Ishikawa cells. Epigenetic modification of the PR promoter has been hypothesized to be another mechanism for regulating PR expression^47–51^. According to a study investigating the methylation status and the expressions of the two PR isoforms in endometrial cancer samples, the specimens with methylated PR-B alleles were negative for the immunohistochemical expression of PR-B, but all of them had unmethylated PR-A alleles^47^. The CpG islands of the PR-B-promoters in PR-B negative cell lines, HEC-1B and KLE, were highly methylated, and treatment with 5-aza-2ʹ-deoxycytidine, a DNA methyltransferase inhibitor, led to an increase in PR-B mRNA along with demethylation of the PR-B promoter in both the cell lines^47,48^. On the contrary, our MSP results indicated that the PR-B promoter was unmethylated prior to the genistein treatment, and the PR-A promoter remained methylated post-treatment in both the cell lines. Thereafter, we focused on c-Jun and c-Fos, which had been listed as candidates for transcription factors using JASPAR, since the mitogen-activated protein kinase (MAPK) pathway and Ras activation have been reported as mechanisms for regulating PR expression^52–55^. A previous report states that C/EBPβ and c-Jun synergize to stimulate a PR promoter-reporter, thereby elevating endogenous PR expression in murine mammary gland^17^, while another report states that C/EBPβ negatively regulates PR expression in human glioblastoma cells^16^. Our results show that genistein lowers C/EBPβ expression, elevates c-Jun expression, and increases phosphorylation of c-Jun and JNK. Genistein has always been considered as a non-specific protein-tyrosine kinase inhibitor^56^. Hence, genistein can maintain long-term PR-inducing effects as a tyrosine kinase inhibitor, regardless of ER status in endometrial cancer.

Finally, the long-term PR-inducing effect of genistein can lead to an improved prognosis in patients with endometrial cancer. Since genistein-induced expression of PR and FOXO1 induces endometrial decidualization, it may be useful for maintenance treatment in young patients with endometrial cancer who wish to preserve their fertility. It has been reported that metformin also promotes PR expression via inhibition of the mammalian target of rapamycin in endometrial cancer cells^57^. A clinical trial has already shown that the combination of medroxyprogesterone and metformin is effective in terms of relapse-free survival and post-treatment conception^58^. Genistein has been found to be safe and non-teratogenic when taken via diet^59^. Therefore, genistein is expected to have an effect similar to metformin in young patients with endometrial cancer.

This study had several limitations. One of the limitations is determining the dosage of genistein intake required to increase PR expression and suppress cell proliferation. In vivo*,* the growth of melanoma tumors implanted into mice was inhibited by 50% when the mice were fed on a genistein-rich diet (1 mg/day). Plasma genistein concentration at the time of tumor removal was 1.1 μM^60^. This is similar to the levels reported in humans who were given two different preparations of unconjugated soy isoflavones, 4.3 and 16.3 μM^61^, but higher than the mean plasma levels detected in Japanese men consuming diets with a high content of soy products^62^. It is difficult to directly compare concentrations used in *vitro* or in *vivo* with circulating concentrations in humans. Epidemiological studies have shown that high genistein intake reduces the frequency of endometrial cancer^63^. Thirteen revised epidemiological studies revealed that isoflavone intake from soy products and legumes is associated with a 19% reduction in endometrial cancer risk^64^. Another limitation is the consideration of stromal PR expression in the tumor microenvironment. In the endometria of premenopausal women, both PR-A and PR-B isoforms are expressed in the epithelial and stromal cells^65^, and both isoforms appear to fluctuate in the cycling endometrium in a cell-specific manner^7^.

In conclusion, our study revealed that genistein induces long-time PR expression and inhibits cell proliferation in an ER-independent manner. Genistein may be a promising therapeutic candidate for improving the prognosis of young patients with endometrial cancer who wish to preserve their fertility.

## Methods

### Patients, specimen collection, and immunohistochemical analysis

Specimens collected from 32 uterine endometrial cancer patients aged < 40 years were evaluated. These patients had undergone dilatation and curettage or hysterectomy at the Department of Obstetrics and Gynecology, Kyoto Prefectural University of Medicine (Kyoto, Japan) between 1997 and 2019. Among them, six patients who had undergone hysterectomy due to ineffective medroxyprogesterone acetate therapy were included. The research protocol was approved by the Institutional Review Board (ERB-C-2412) and performed in accordance with relevant guidelines and regulations. Informed consent was obtained from all the patients prior to the study. Immunohistochemical staining was performed, as previously described^66,67^. Furthermore, the level of PR expression was assessed by H-score, a commonly used method to measure the strength of ER- and PR-staining that evaluates the intensity of staining and the percentage of cells stained at each intensity in a semi-quantitative manner. Intensities were scored as 0 (no staining), 1 (weak staining), 2 (moderate staining), and 3 (strong staining). The H-score was calculated using the following algorithm: H-score = ∑ (i + 1) × Pi (where i and Pi represent the intensity of staining and percentage of cells at each intensity, respectively). We used the quantification method to analyze the relation of H-score to survival rate, thereby categorizing the H-score into 2 groups, namely 1–100 and > 100.

### Antibodies

The mouse monoclonal antibodies, namely anti-PR (sc-166169), anti-ERβ (sc-53494), anti-c-Jun (sc-74543), anti-p-c-Jun (sc-822), anti-JNK (sc-7345), anti-p-JNK (sc-6254), anti-C/EBPβ (sc-7962), and rat monoclonal antibody anti-ERα (sc-53493) were purchased from Santa Cruz Biotechnology (Dallas, TX, USA). The rabbit polyclonal antibodies, including anti-phospho-cdc2 (Tyr15) (#9111), anti-cdc2 (#9112), anti-cleaved caspase 3 (Asp175) (#9661), anti-caspase 3 (#9662); rabbit monoclonal antibodies, including anti-phospho-cdc25C (Ser216) (#4901), anti-cdc25C (#4688), anti-phospho-histone H3 (Ser10) (#3377), anti-glyceraldehyde 3-phosphate dehydrogenase (anti-GAPDH) (#2118), anti-PR A/B (#3153), anti-FOXO1 (#2880), and anti-IGFBP1 (#31025) antibodies; and the anti-rabbit (#7074) and anti-mouse (#7076) IgG, horse radish peroxidase (HRP)-linked antibodies were obtained from Cell Signaling Technology (Beverly, MA, USA). The mouse monoclonal anti-Ki-67 (M7240) was purchased from Dako (Agilent; Santa Clara, CA, USA). All antibodies were used at the concentrations recommended by the manufacturers.

### Cell lines and cell culture

The human uterine endometrial cancer cell line Ishikawa was provided by the Cell Resource Center for Biomedical Research (Institute of Development, Aging and Cancer, Tohoku University, Sendai, Japan). The human uterine endometrial cancer cell line KLE was purchased from the American Type Culture Collection (ATCC^Ⓡ^; Manassas, VA, USA). We used the ATCC^Ⓡ^ human short-tandem repeat (STR) profiling cell authentication service to confirm that our Ishikawa cell line sample was a match to the STR profile for this cell line listed on the ExPASy website, and our KLE cell line sample was an exact match to the ATCC cell line CRL-1622 (KLE). The Ishikawa cells were maintained in Minimum Essential Medium (MEM; Nacalai Tesque, Kyoto, Japan) with sodium pyruvate, and the KLE cells were cultured in Dulbecco’s modified Eagle’s medium (DMEM)/Ham’s F-12 (Nacalai Tesque). Each medium was supplemented with 10% fetal bovine serum (FBS; Biowest, Nuaille, France) and penicillin–streptomycin (Nacalai Tesque). All cells were cultured at 37 °C in a humidified atmosphere 5% CO_2_.

### Reagents

The isoflavone metabolites, genistein, daidzein, glycitein, and equol were purchased from Cayman Chemical Company (Ann Arbor, MI, USA). The Ishikawa and KLE cells were incubated in phenol-red free MEM and DMEM/Ham’s F-12, respectively, supplemented with 20 μM of each isoflavone metabolite in a humidified atmosphere with 5% CO_2_.

### Cell proliferation assay

Cells were seeded in 96-well plates with normal growth medium. After 24 h, dimethyl sulfoxide (DMSO; control) or various doses of genistein were added. The cells were cultured and treated in 12 replicates. Cell viability was examined every 1 or 2 days using the 2-(2-methoxy-4-nitrophenyl)-3-(4-nitrophenyl)-5-(2,4-disulfophenyl)-2H-tetrazolium, a monosodium salt (WST-8) assay (Nacalai Tesque). Each experiment was performed in triplicate.

### Flow cytometry analysis

Cells were seeded in 6-well plates and incubated for 7 days while changing the medium every 2 days. Cells were permeabilized with 0.1% Triton-X100 and the nuclei were then stained with propidium iodide (PI). The DNA content was measured using a fluorescence-activated cell sorting (FACS) Caliber cytometer (BD Biosciences, Franklin Lakes, NJ, USA) and analyzed with ModFit LT (Verty Software, Topsham, ME, USA) and Cell Quest software packages (BD Biosciences).

### RNA extraction and qPCR analysis

RNA extraction and qPCR analysis were performed, as described previously^68^. Total RNA (1 μg) was extracted from cultured cells using RNeasy Mini kit (QIAGEN, Venlo, Netherlands), and cDNA was synthesized from 1 μg of RNA using ReverTra Ace qPCR RT kit (Toyobo, Osaka, Japan). The qPCR analysis was performed using CFX Connect Real-Time PCR Detection System (Bio-Rad, Hercules, CA, USA). The cDNA samples (1 μL) generated from the total RNA from of the cells were mixed into 20 μL reaction mixtures containing SYBR qPCR Thunderbird master mix (Toyobo) and 0.3 μM of each primer. The following primers were designed with Primer3Plus free software and purchased from Invitrogen: ERα 5′- GGTTGGTGGCTGGACACATA-3′ (forward) and 5′-CGCTACTGTGCAGTGTGCAA-3′ (reverse), ERβ 5′-GAGCTGTTGGATGGAGGTGT-3′ (forward) and 5′- CTTCTACGCATTTCCCCTCA-3′ (reverse), PR-AB 5′-AGAAAAGGACAGCGGACTGC-3′ (forward) and 5′-CAAACAGGCACCAAGAGCTG-3′ (reverse), PR-B 5′-GAAAAAGTCGGGAGATAAAGGAGC-3′ (forward) and 5′-AAGAGAGTTCTCCAACTTCTGTCC-3′ (reverse), C/EBPβ 5′-GAAGACCGTGGACAAGCACA-3′ (forward) and 5′-CGTGAGCTCCAGGACCTTGT-3′ (reverse), and GAPDH 5′-GCACCGTCAAGGCTGAGAAC-3′ (forward) and 5′-ATGGTGGTGAAGACGCCAGT-3′ (reverse). The mRNA levels of the target genes were quantified using the comparative method (∆∆CT method) and normalized to GAPDH expression.

### Western blot analysis

Western blot analysis was performed, as described previously^68^. Cells were washed thrice with phosphate-buffered saline and lysed in radioimmunoprecipitation assay (RIPA) buffer (Nacalai Tesque). Cell lysates (10–20 μg) were heated in sodium dodecyl sulfate (SDS) sample buffer (125 mM Tris–HCl, pH 6.8, 4% SDS, 25% glycerol, 10% 2-mercaptoethanol, 0.05 mM phenylmethanesulfonyl fluoride, and 0.004% bromophenol blue), separated by 10% or 15% e-PAGEL (Atto Corp, Tokyo, Japan) according to the manufacturer’s recommendations, and transferred onto polyvinylidene fluoride (PVDF) immuno-blot membranes (Bio-Rad). The membranes were blocked with PVDF Blocking Reagent for Can Get Signal (Toyobo) for 1 h at 20–25 °C and incubated with the appropriate primary antibody in Can Get Signal Solution 1 (Toyobo) overnight at 4 °C. After washing, the membranes were incubated with the appropriate secondary antibody for 1 h at 20–25 °C. The signal was visualized by Chemi-Lumi One (Nacalai Tesque) and analyzed by ChemiDoc XRS + system with Image Lab software (Bio-Rad).

### DNA extraction and sodium bisulfite sequencing

Genomic DNA was extracted from the cultured cells using DNAiso Reagent (Takara Bio Inc., Shiga, Japan), and 10 ng of the isolated DNA was subjected to sodium bisulfite conversion using EpiTect^Ⓡ^ Fast DNA Bisulfite Kit (QIAGEN), according to the manufacturer’s instructions. The converted DNA was eluted from DNA affinity columns, and 2 μL was used for subsequent PCR analyses.

### The MSP analysis

The PCR analysis was performed using Premix Ex Taq™ Hot Start Version (Takara Bio Inc.) and a hot start procedure. The following primers were used for this process: PR-A-W 5′-ACGGGCTACTCTTCCCTCG-3′ (forward) and 5′-TGGAATATGCGCCCTCCACG-3′ (reverse), PR-A-U 5′-ATGGGTTATTTTTTTTTTG-3′ (forward) and 5′-TAAAATATACACCCTCCACA-3′ (reverse), PR-A-M 5′-ACGGGTTATTTTTTTTTCG-3′ (forward) and 5′-TAAAATATACGCCCTCCACG-3′ (reverse), PR-B-W 5′-TGACTGTCGCCCGCAGTACG-3′ (forward) and 5′-CGGCAATTTAGTGACACGCG-3′ (reverse), PR-B-U 5′-TGATTGTTGTTTGTAGTATG-3′ (forward) and 5′-CAACAATTTAATAACACACA-3′ (reverse), and PR-B-M 5′-TGATTGTCGTTCGTAGTACG-3′ (forward) and 5′-CGACAATTTAATAACACGCG-3′ (reverse) (Supplementary Fig. 2).

### Animal model

Female BALB/c nu/nu mice (4-weeks-old) were purchased from Shimizu Co., Ltd. (Kyoto, Japan) and housed under specific pathogen-free conditions, as previously reported. Ishikawa cells (8.5 × 10^6^ cells per mouse) were inoculated subcutaneously into the flanks of the mice (8-weeks-old). The resulting tumor volumes were calculated using the formula: 1/2 × (length) × (width)^2^. After establishment of a palpable tumor (approximately 30 mm^3^), the mice were randomly divided into control and genistein groups (n = 6 for each). Subsequently, for 4 weeks, the mice in the genistein group received abdominal injections of genistein (90 mg/kg body weight) dissolved in DMSO on alternate days. Mice in the control group were treated with an equal volume of the vehicle. Tumor volume and body weight were sequentially measured once every 2 or 3 days for 4 weeks. The ratio of tumor volume was calculated by comparing with the baseline value recorded at the beginning of the drug treatment. After 8 weeks of inoculation, the mice were euthanized, and the tumors were collected. All experiments and procedures were approved by the Institutional Animal Care Use Committee of Kyoto Prefectural University of Medicine (M2020-21) and performed in accordance with the guidelines of the United Kingdom Coordinating Committee on Cancer Research and the ARRIVE guidelines.

### Statistical analysis

The mean and standard errors between the two groups were compared using a two-tailed unpaired Student’s *t*-test. Comparisons of > 3 groups were performed using one-way analysis of variance (ANOVA) with post-hoc Tukey’s multiple comparison test and two-way ANOVA with Bonferroni post-hoc test. Disease-free survival was estimated according to the Kaplan–Meier method, and the log-rank test was used to calculate statistical significance. These statistical analyses were performed using GraphPad Prism ver. 5.04 (GraphPad Software; San Diego, CA, USA). The *P* values < 0.05 were considered to be statistically significant.

## Supplementary Information

Supplementary Information.

## Data Availability

The datasets generated and analyzed during the current study are available from the corresponding author upon reasonable request.
