# Supplementary material for: Genistein induces long-term expression of progesterone receptor regardless of estrogen receptor status and improves the prognosis of endometrial cancer patients
Source: Sci Rep. 2022 Jun 18;12:10303. doi: 10.1038/s41598-022-13842-6 (PMC9206647; doi:10.1038/s41598-022-13842-6)
Supplement: Supplementary file 1 — Supplementary Information. [file 41598_2022_13842_MOESM1_ESM.docx]

**Supplementary Information**

**Genistein induces long-term expression of progesterone receptor regardless of estrogen receptor status and improves the prognosis of endometrial cancer patients**

**Authors:** Kaori Yoriki, Taisuke Mori, Kohei Aoyama, Yosuke Tarumi, Hisashi Kataoka, Tetsuya Kokabu, Jo Kitawaki

**Supplementary Figure 1**

**(A)**

**
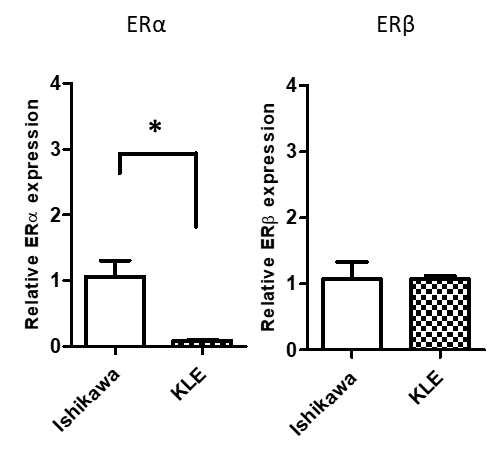

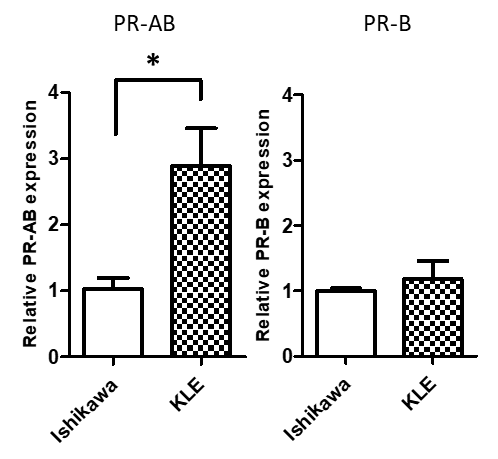
**

**(B)**

**
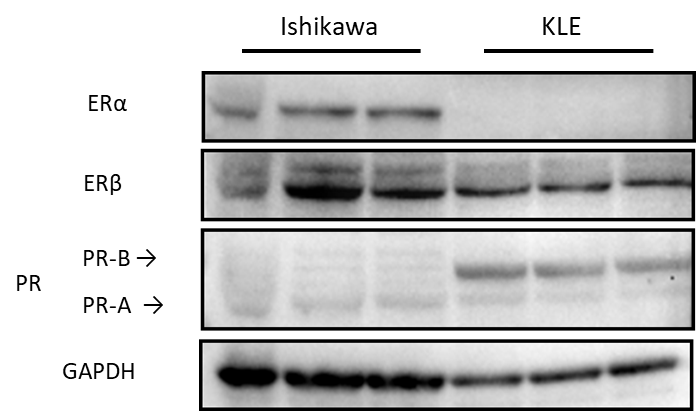
**

**
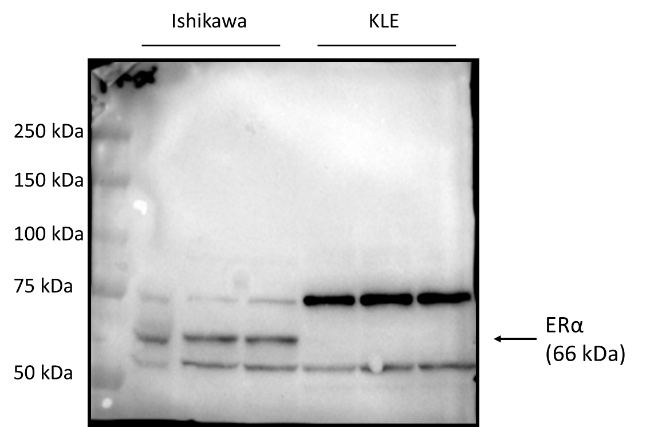
　
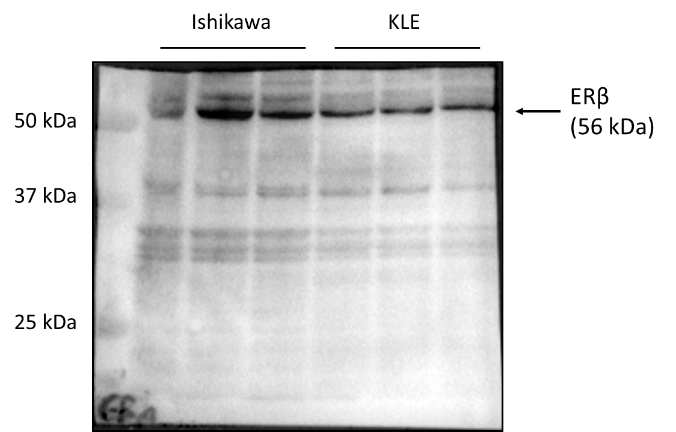
**

**
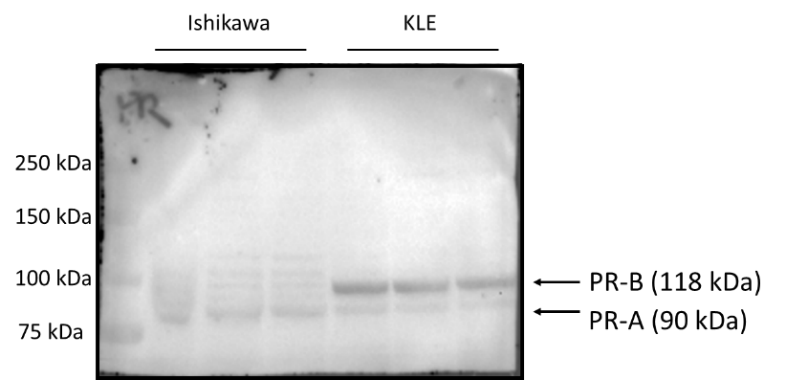

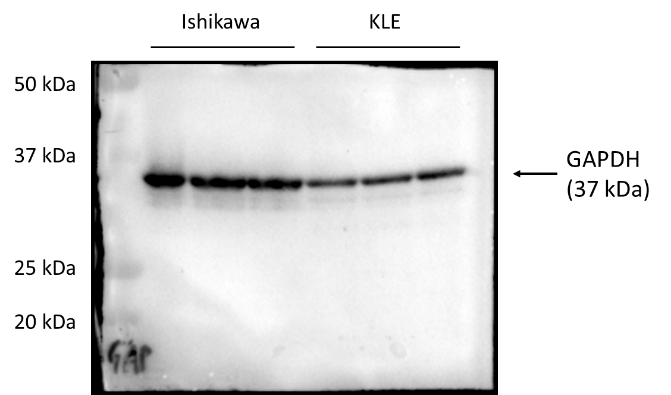
**

**Supplementary Fig. 1:** The expression of estrogen receptor (ER)α, ERβ, progesterone receptor (PR)-AB, and PR-B was examined by quantitative polymerase chain reaction (A) and western blot analysis (B) in Ishikawa and KLE cells.

**Supplementary Figure 2**


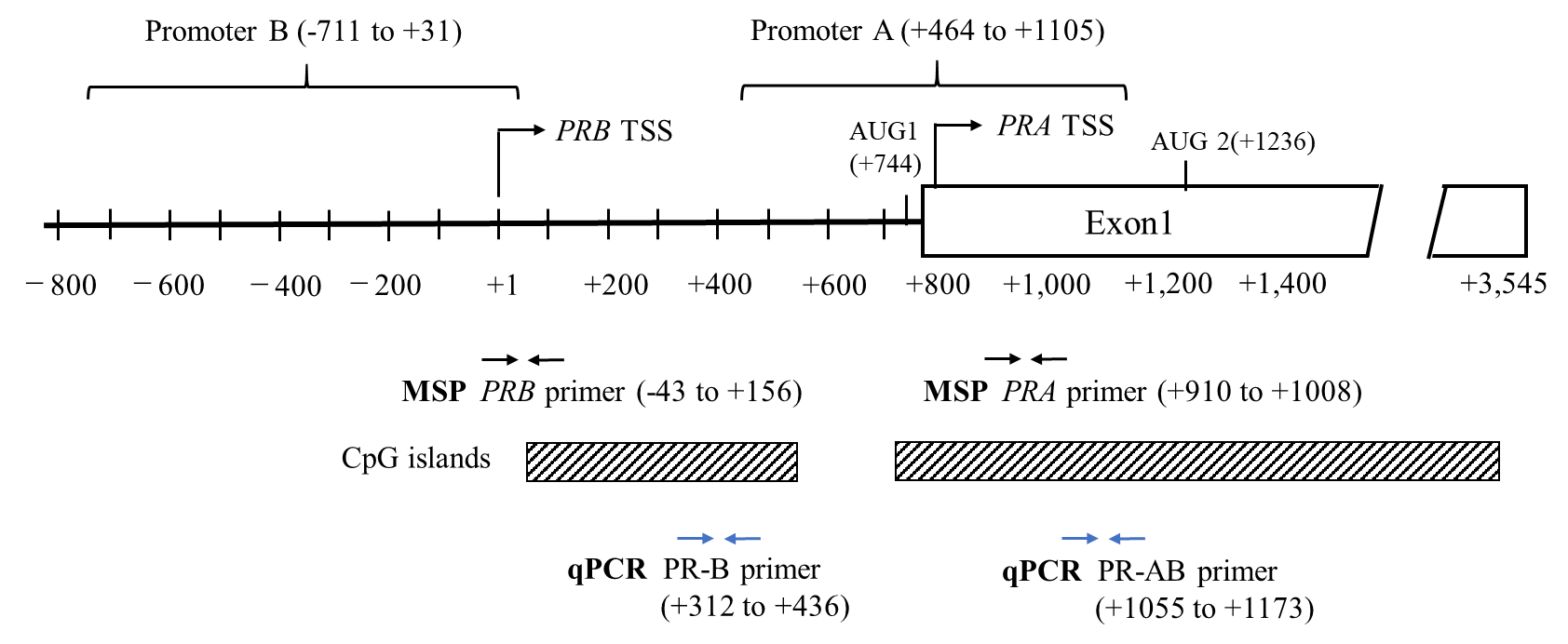


**Supplementary Fig. 2:** Positions and orientation of methylation-specific polymerase chain reaction (MSP) and quantitative polymerase chain reaction primers (qPCR). The-NH2-terminal regions A and B contain the two AUGs (AUG1 at +744 and AUG2 at +1236), which are the translational start sites of progesterone receptor (PR)-B and PR-A.

**Supplementary Figure 3**

**
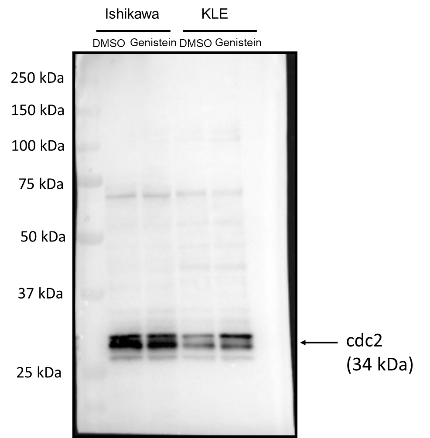

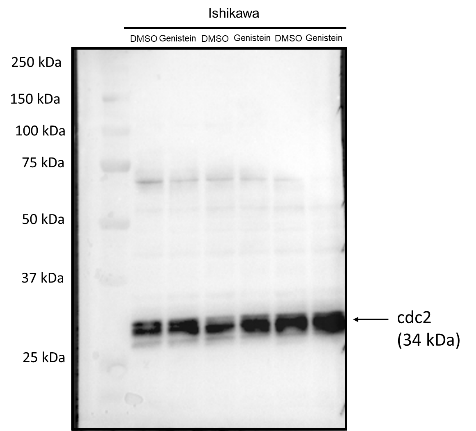

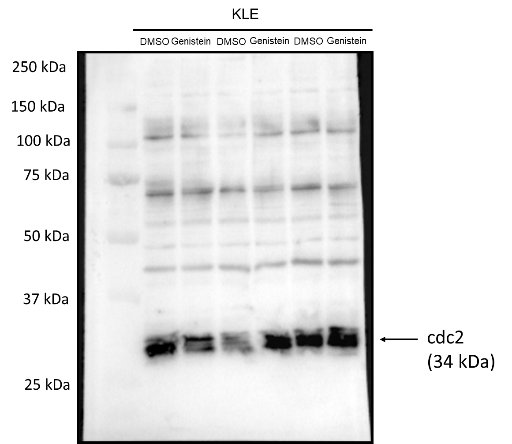
**

**
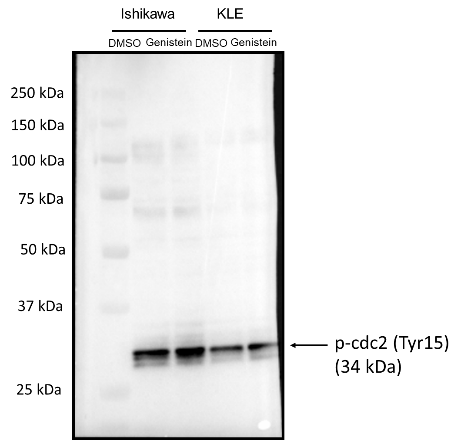

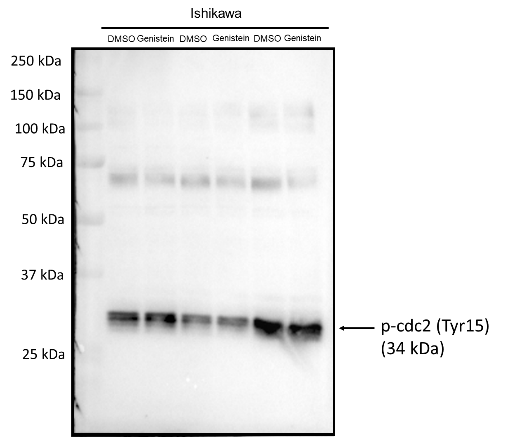

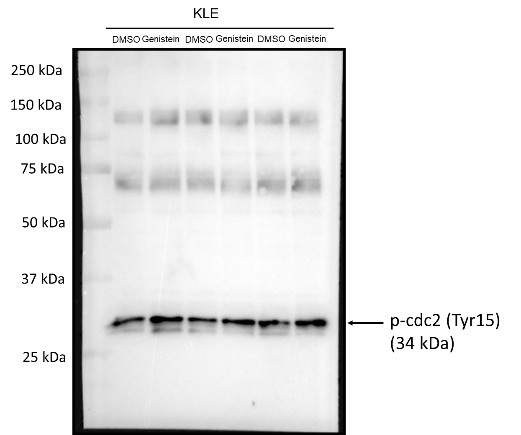
**

**
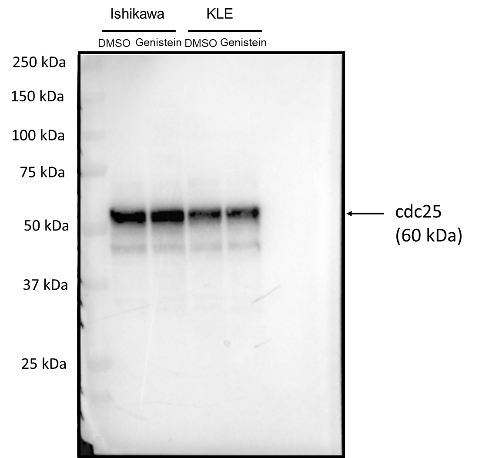

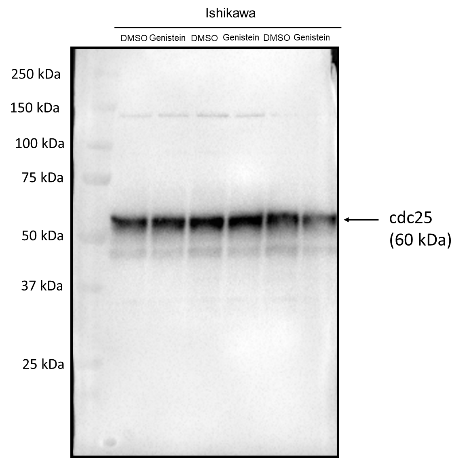

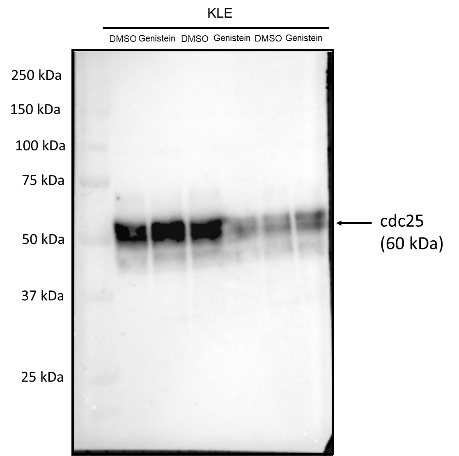
**

**
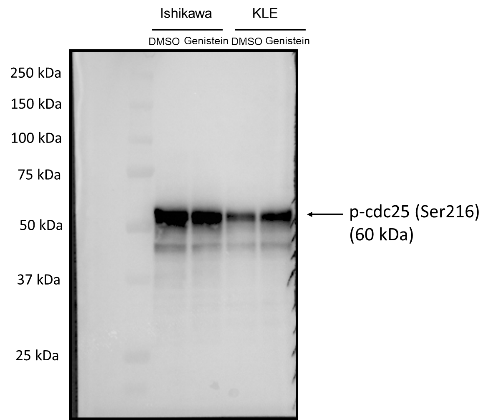

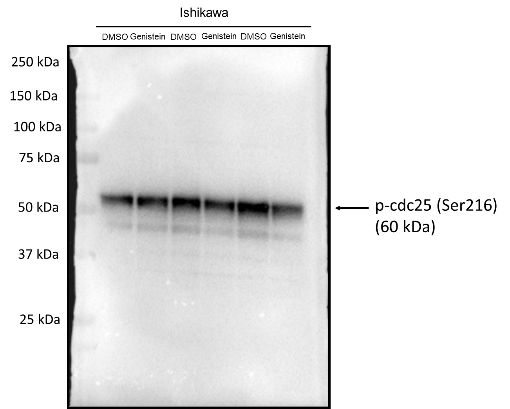

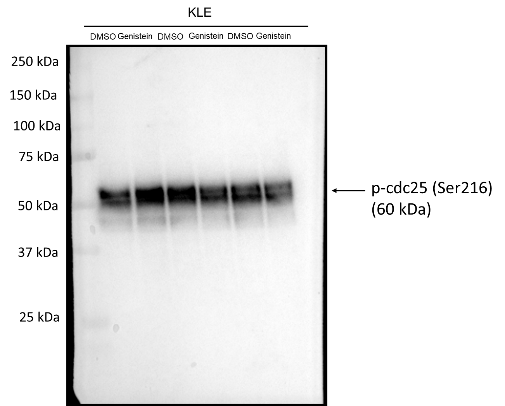
**

**
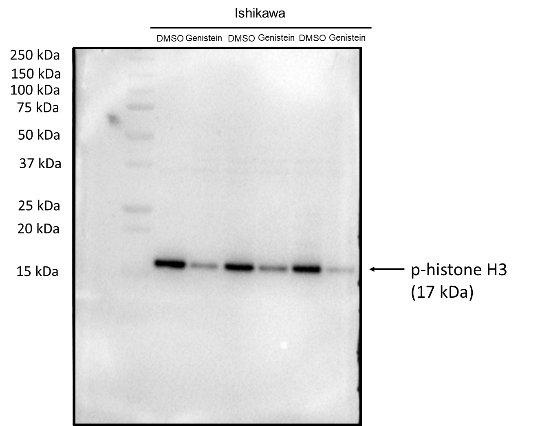

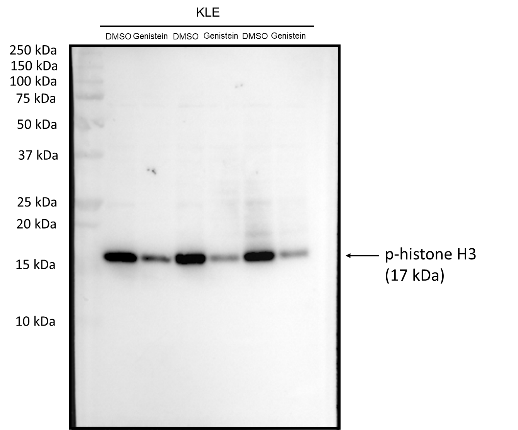
**

**
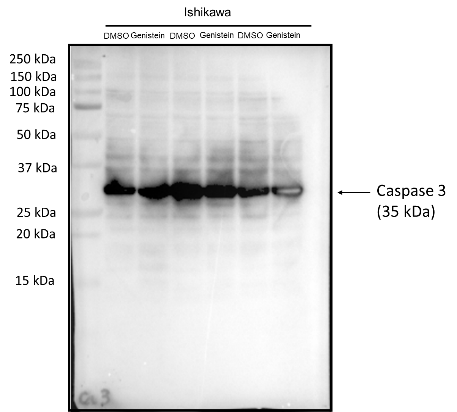

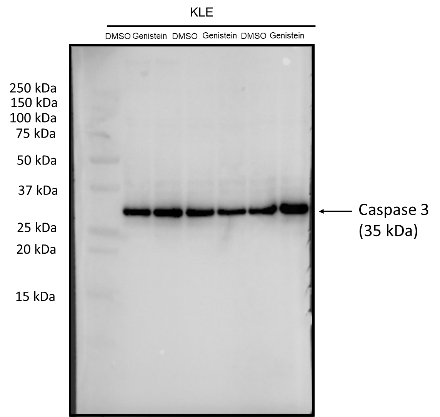
**

**
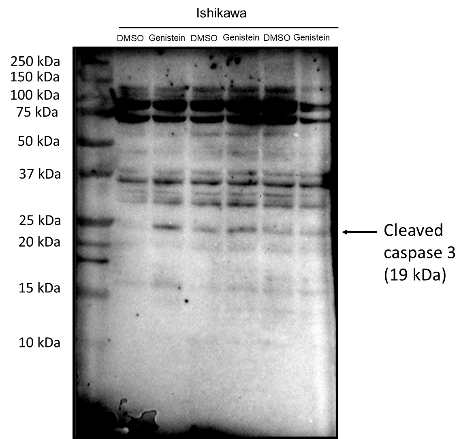

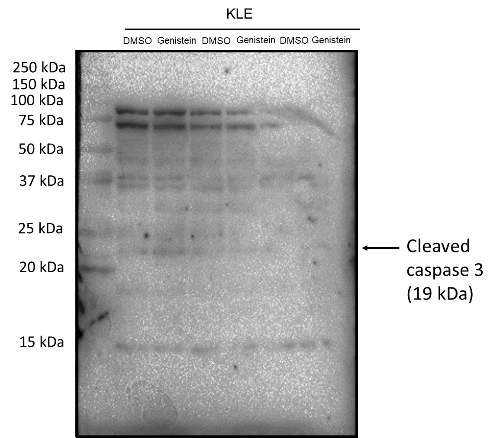
**

**
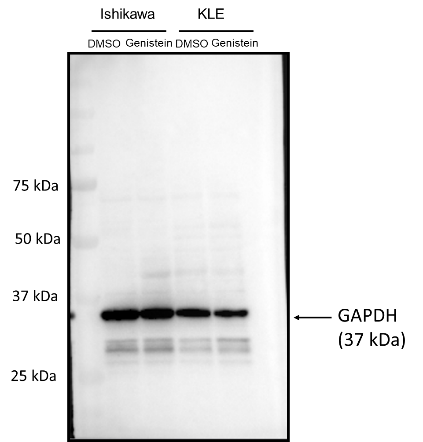

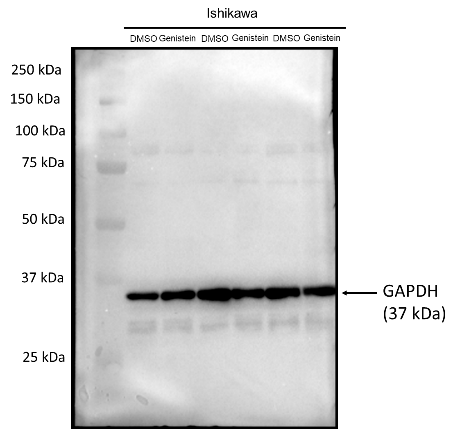

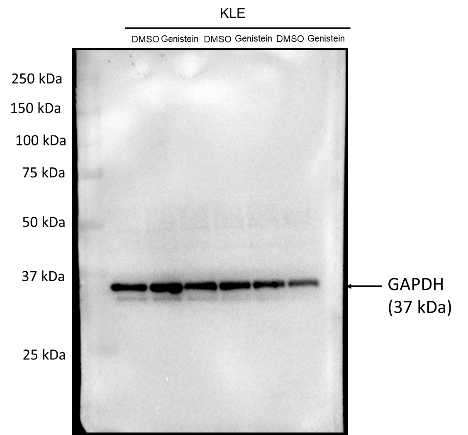
**

**Supplementary Fig. 3:** Full-length western blots of cdc2, p-cdc2 (Tyr15), cdc25, p-cdc25 (Ser216), p-histone H3, caspase 3, cleaved caspase 3, and GAPDH protein expression for Figure 2D.

**Supplementary Figure 4**

**(A)**

**
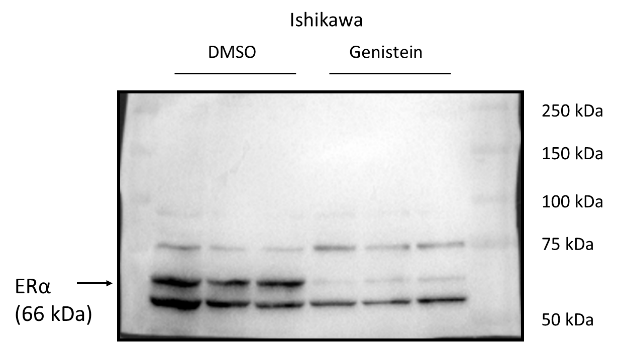

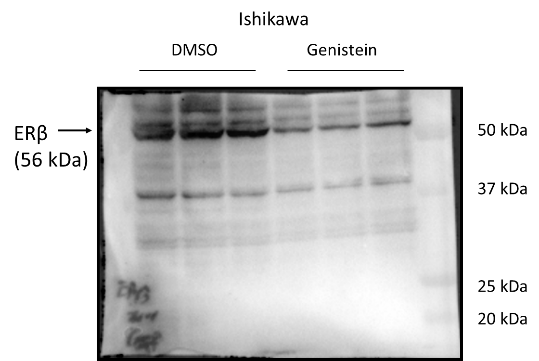
**

**
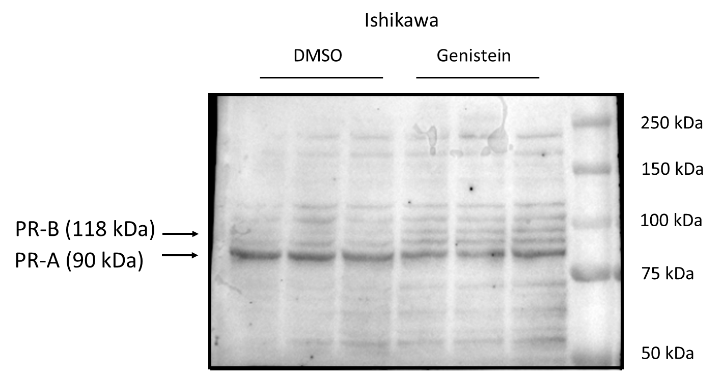

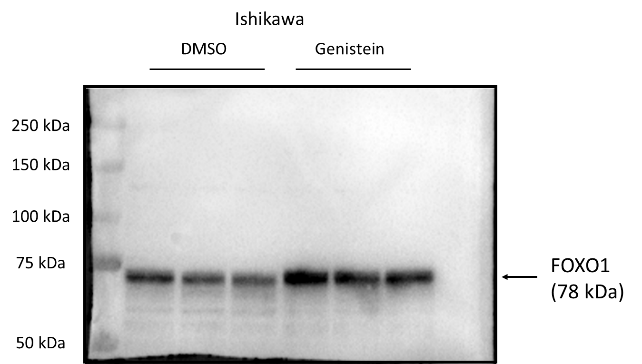
**

**
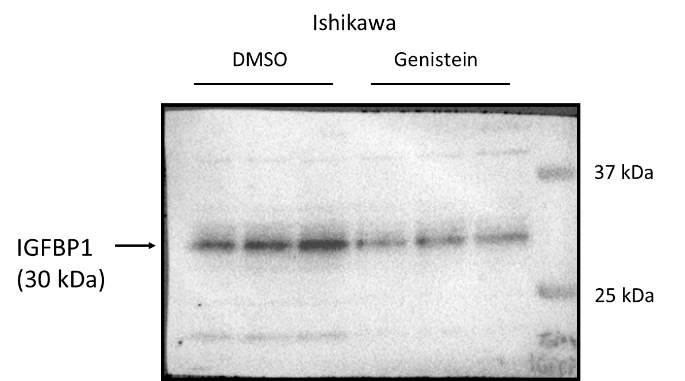

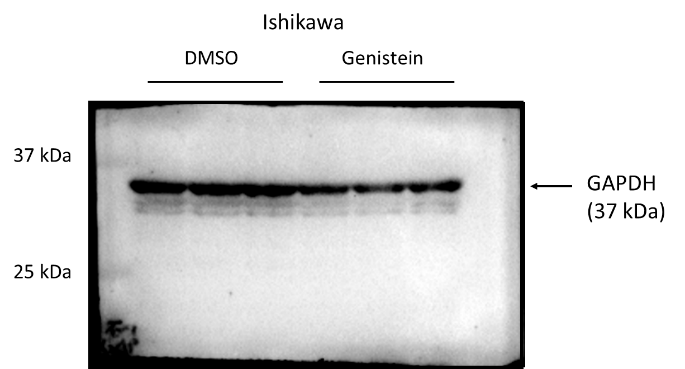
**

**
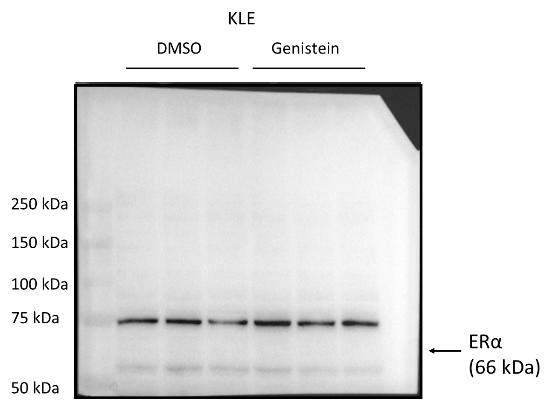

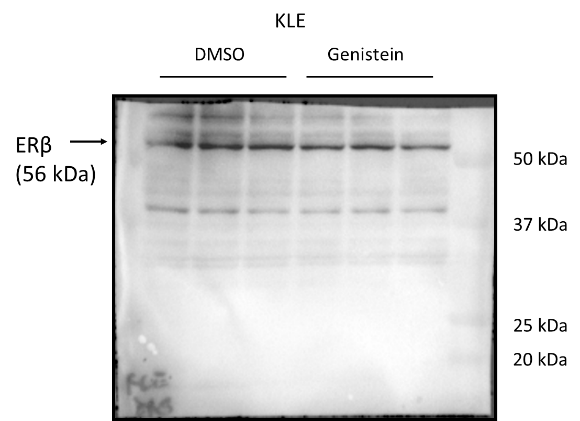
**

**
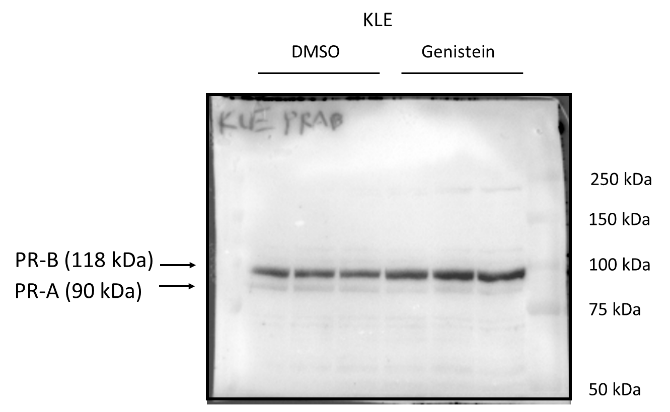

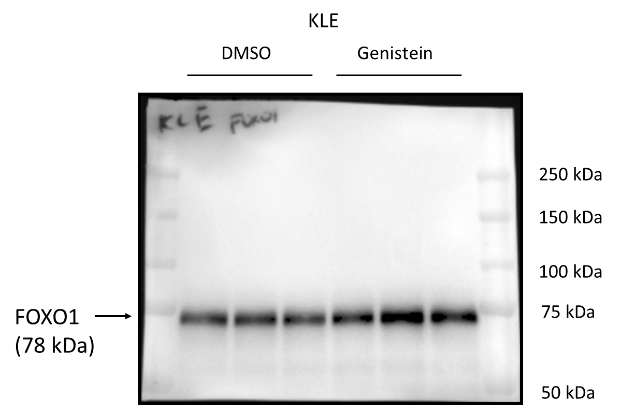
**

**
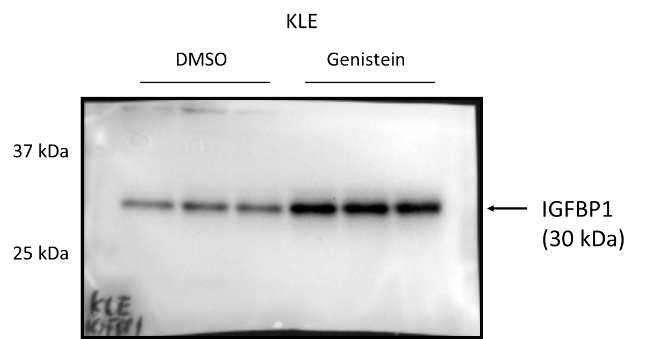

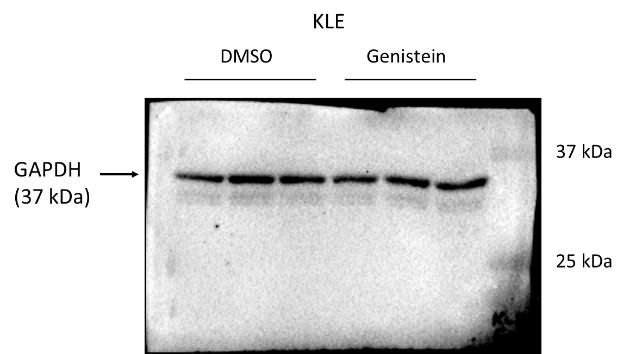
**

**(B)**

**
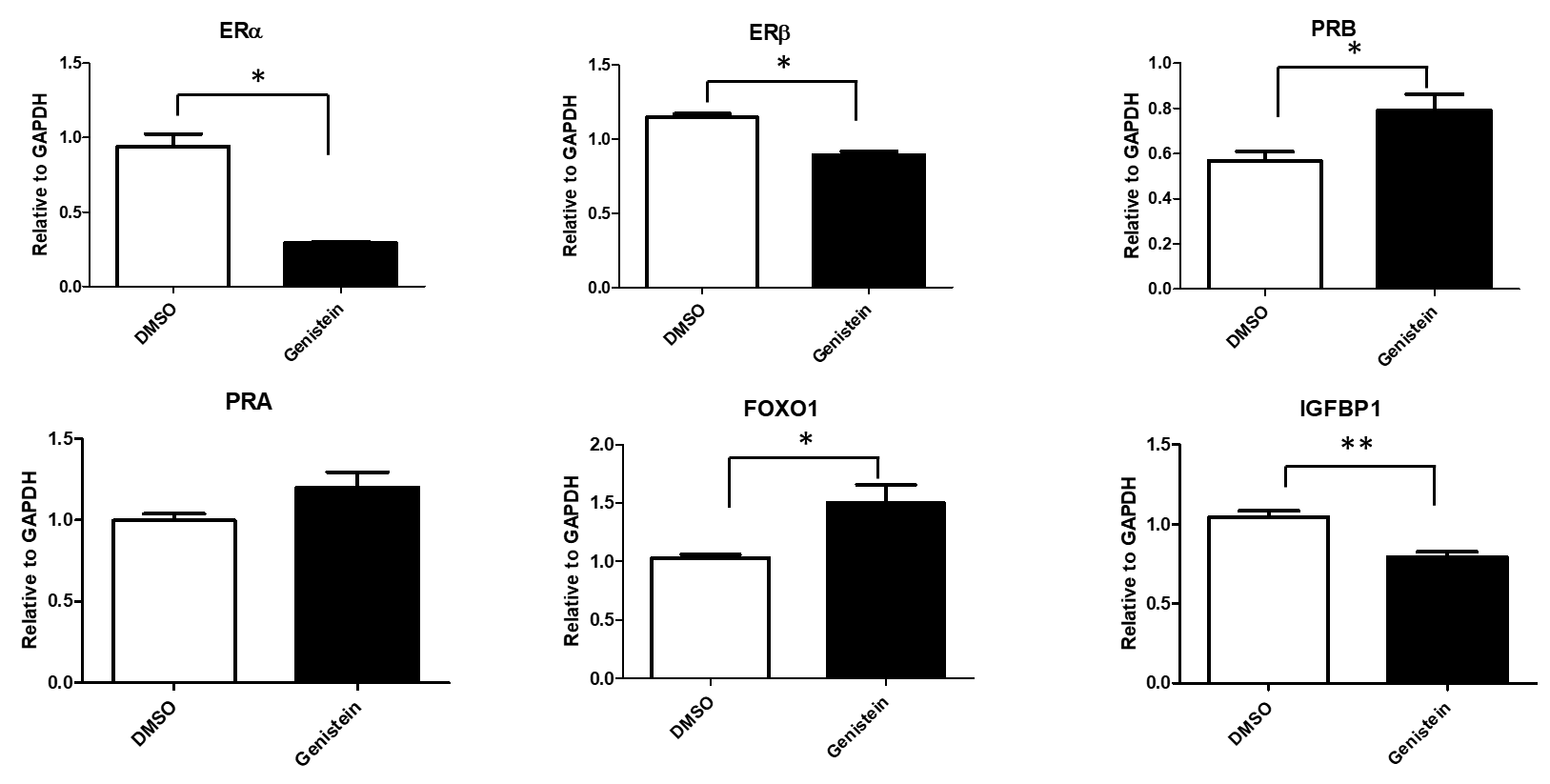
**

**(C)**

**
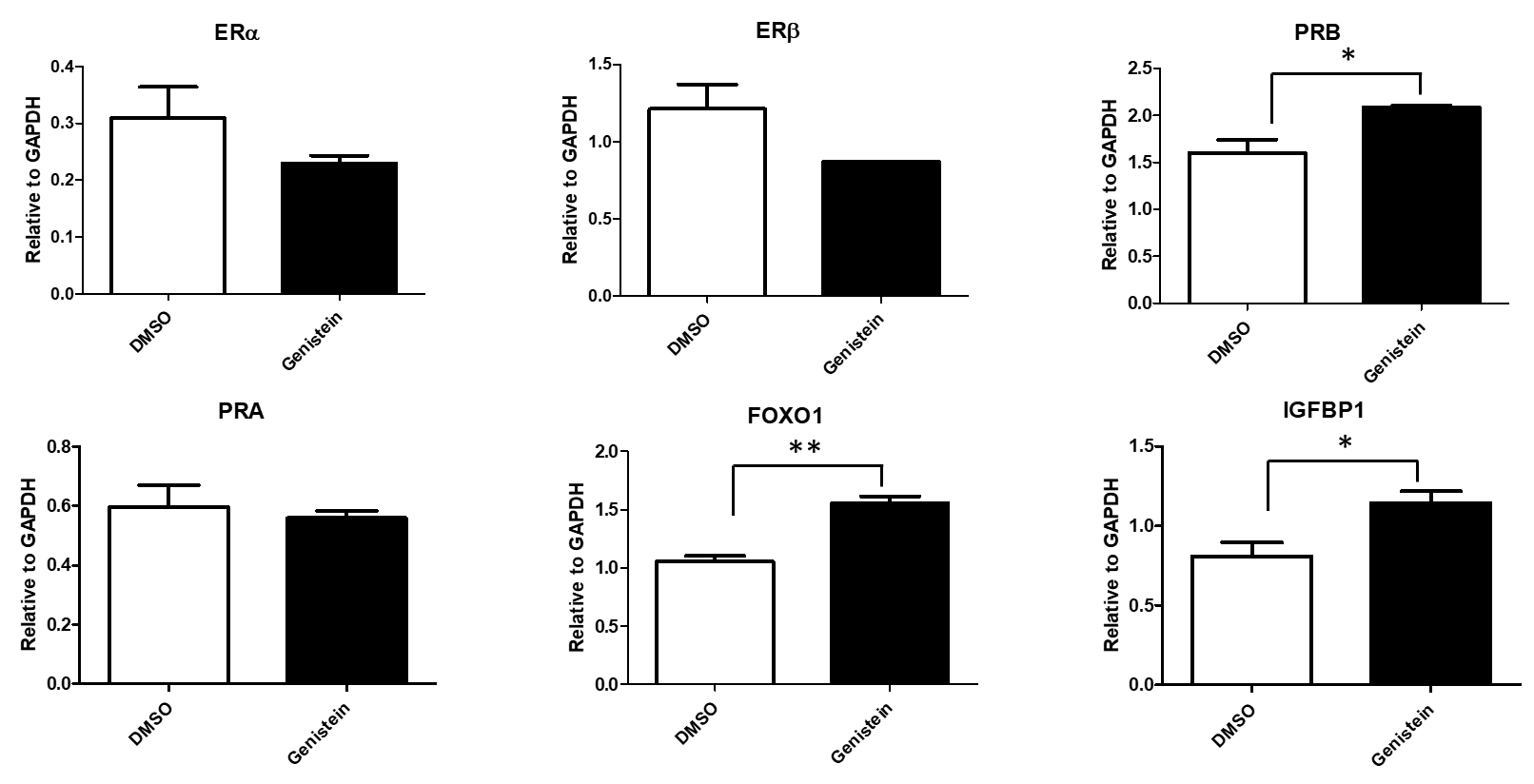
**

**Supplementary Fig. 4:** Full-length western blots of ERα, ERβ, PR-A, PR-B, FOXO1, IGFBP1, and GAPDH protein expression for Figure 3F (A) and the quantification of the western blot analysis in Ishikawa cells (B) and KLE cells (C). *P* values are based on Student’s *t*-test. **P* < 0.05; ***P* < 0.01.

**Supplementary Figure 5**

**
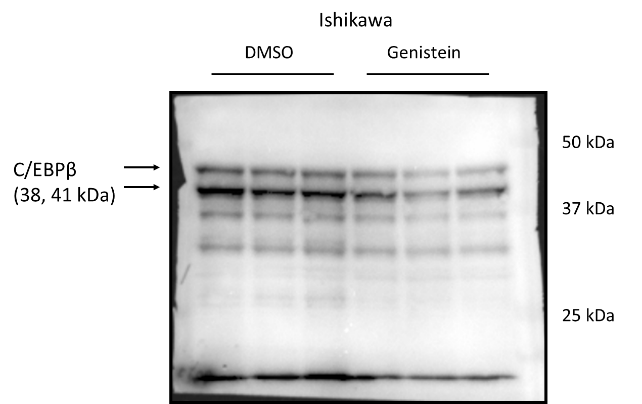

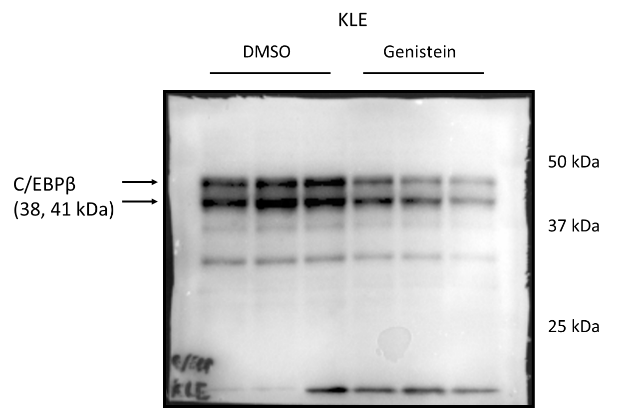
**

**
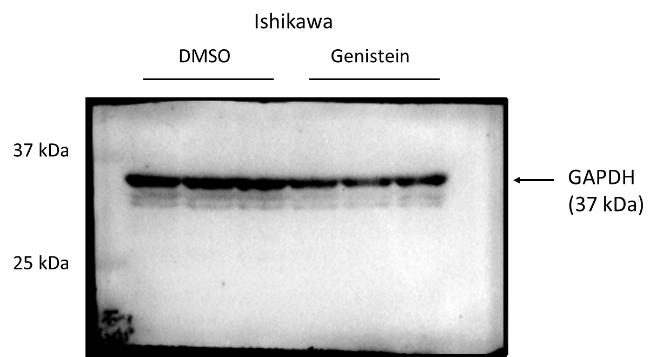

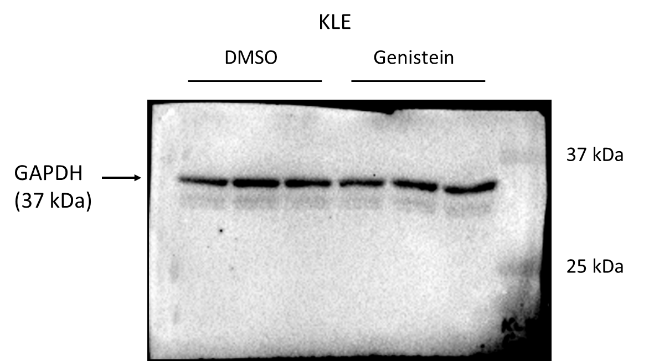
**

**Supplementary Fig. 5:** Full-length western blots of C/EBPβ and GAPDH protein expression for Figure 4B.

**Supplementary Figure 6**

**
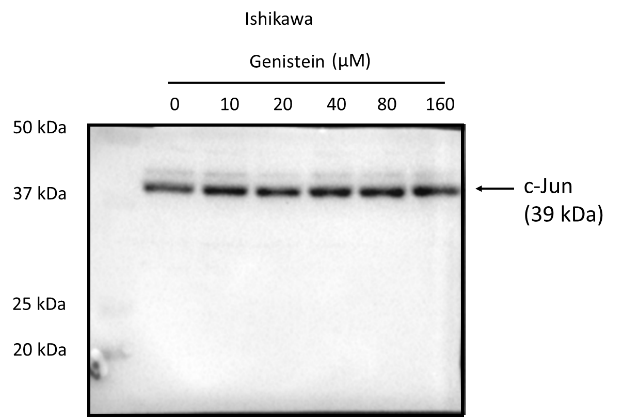

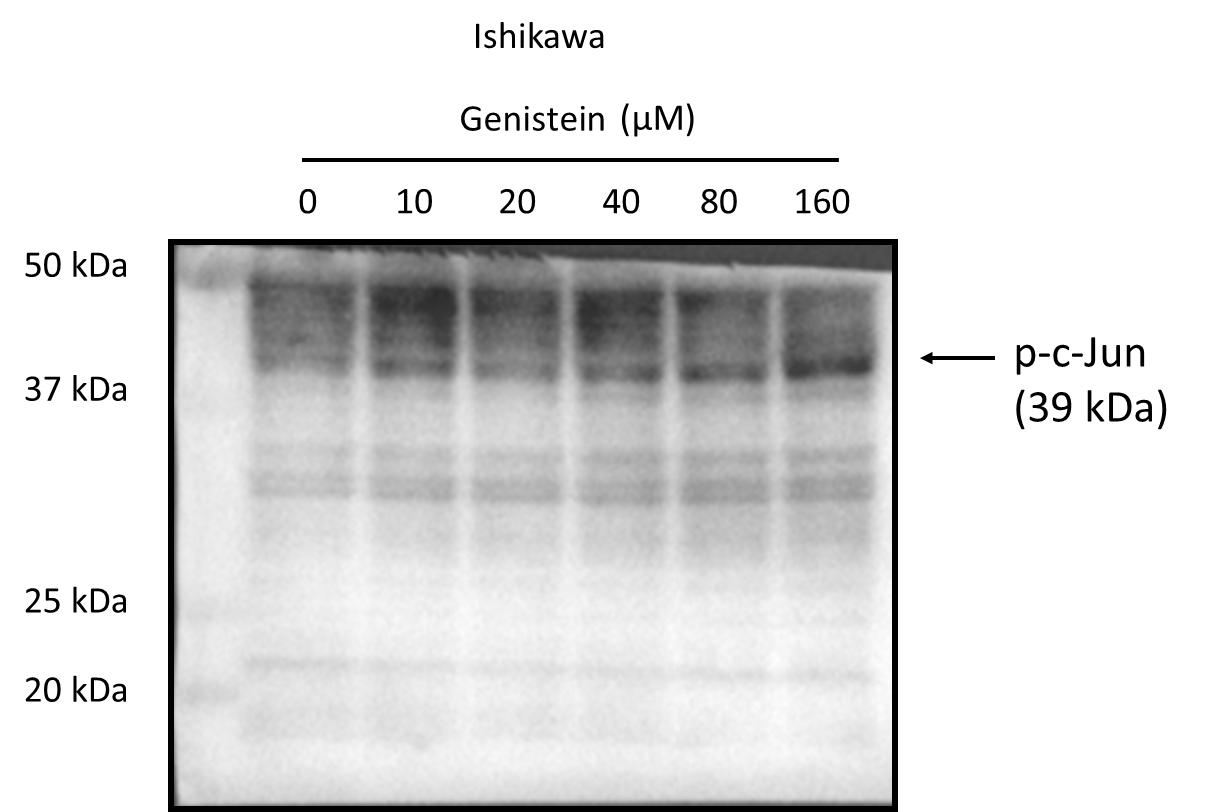
**

**
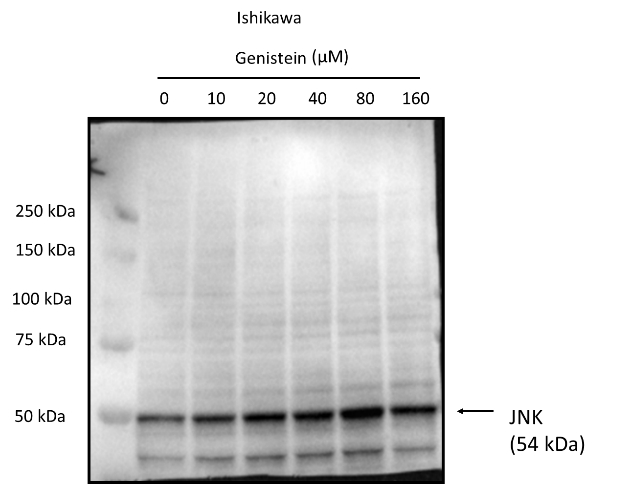

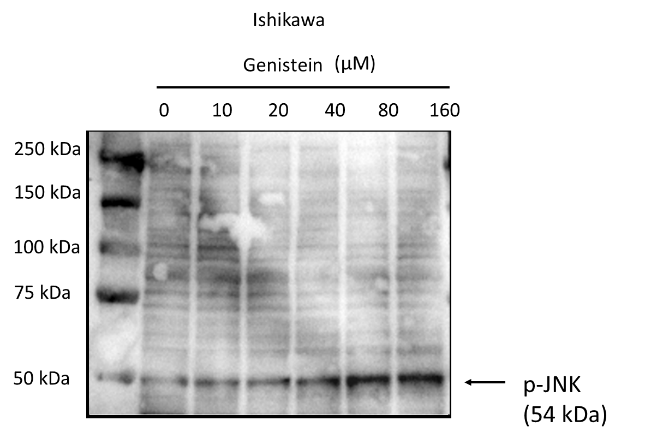
**

**
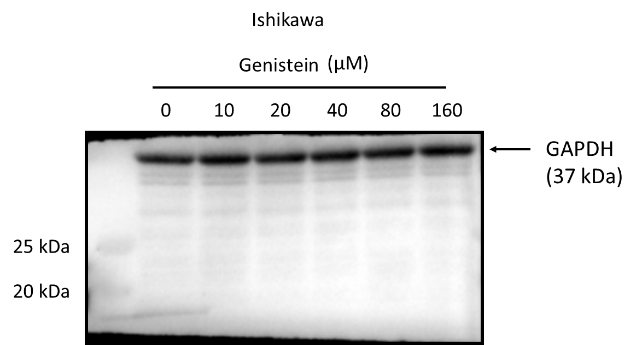

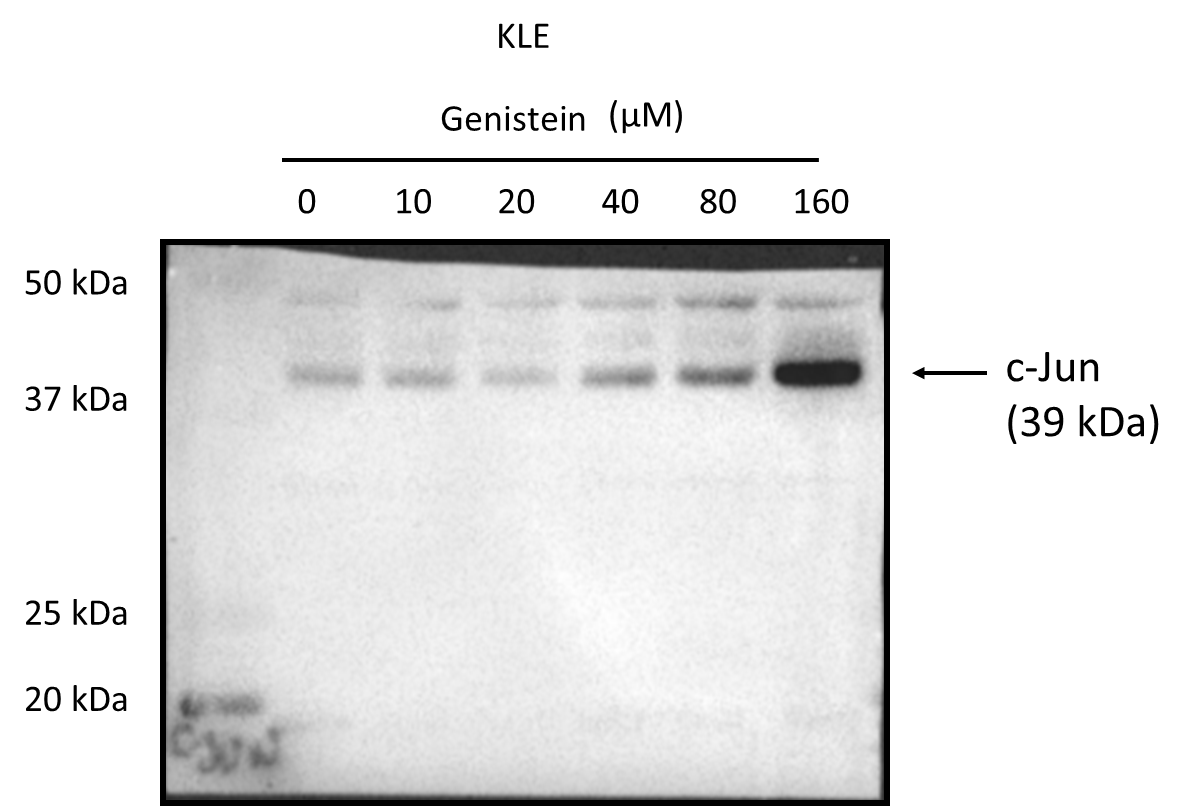
**

**
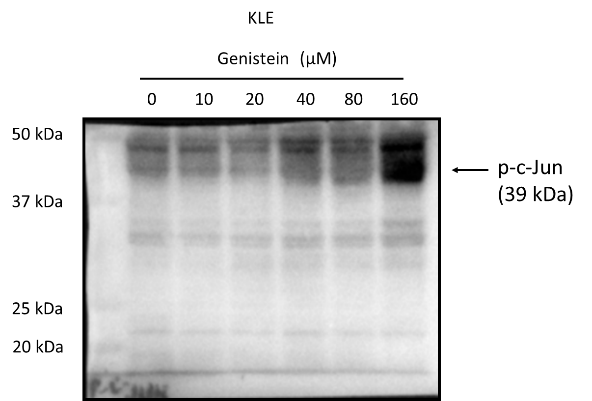

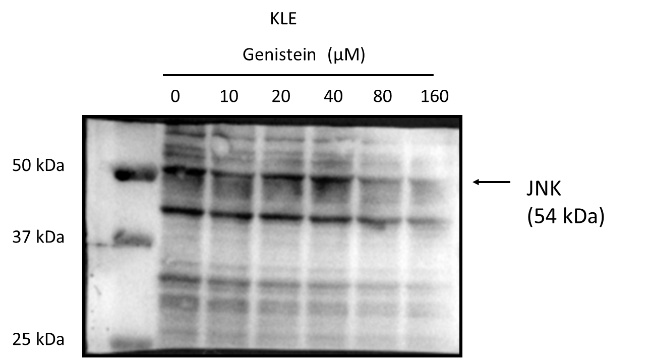
**

**
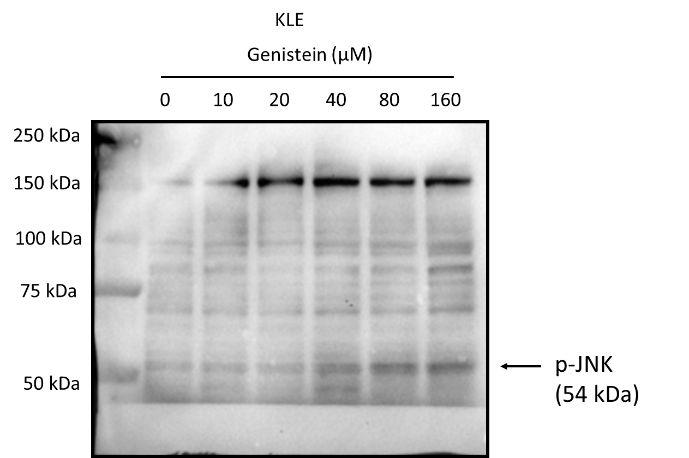

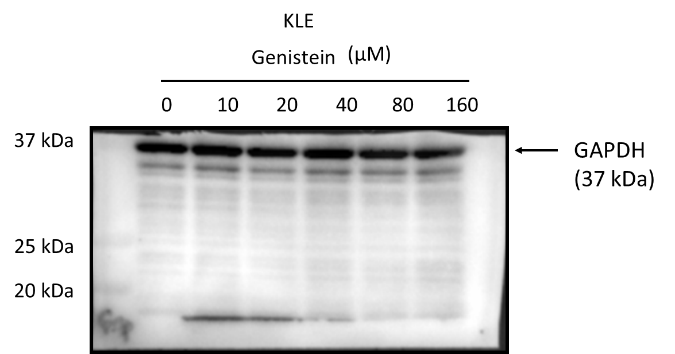
**

**Supplementary Fig. 6:** Full-length western blots of c-Jun, p-c-Jun, JNK, p-JNK, and GAPDH protein expression for Figure 4D.

**Supplementary Figure 7**

**
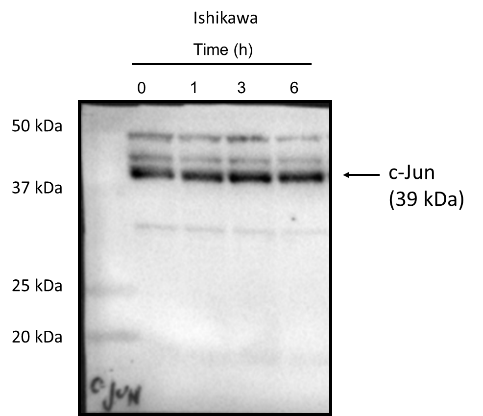

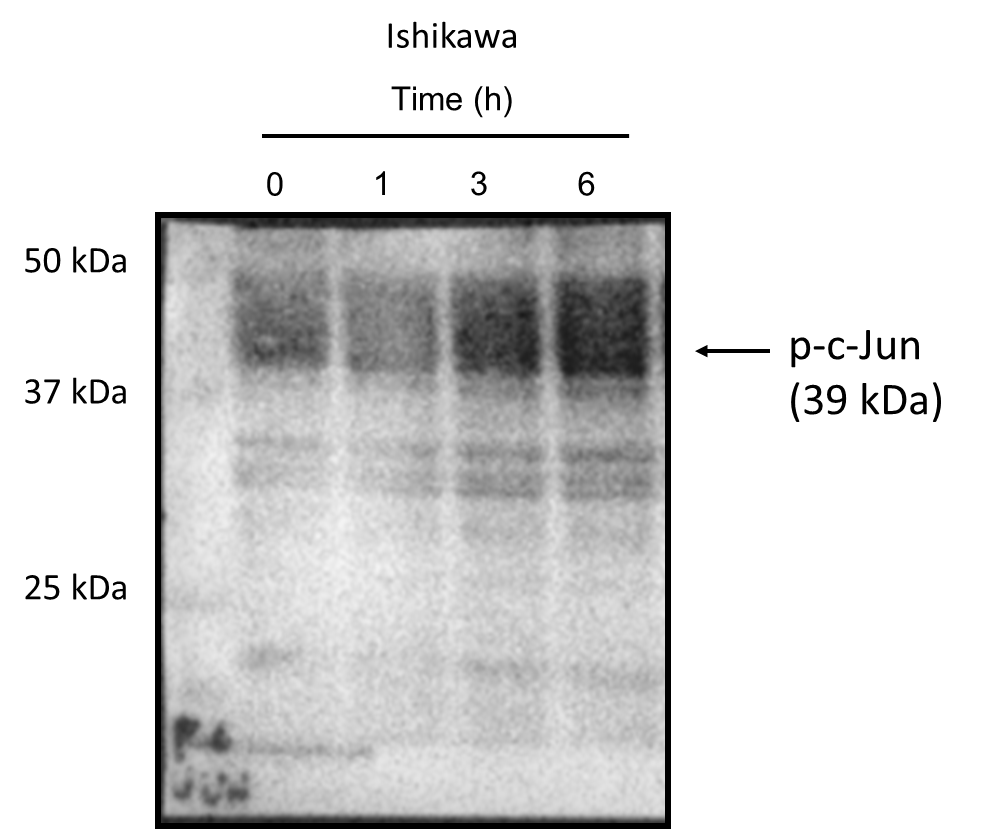

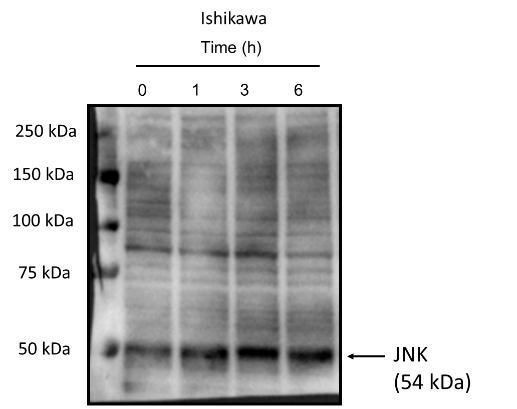
**

**
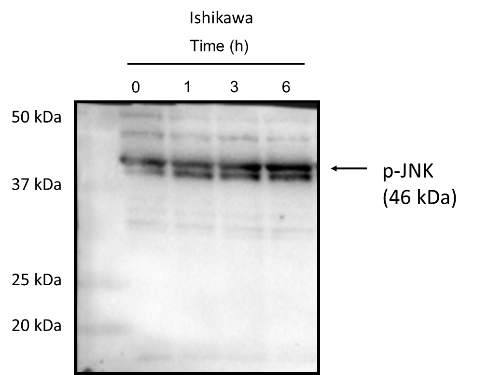

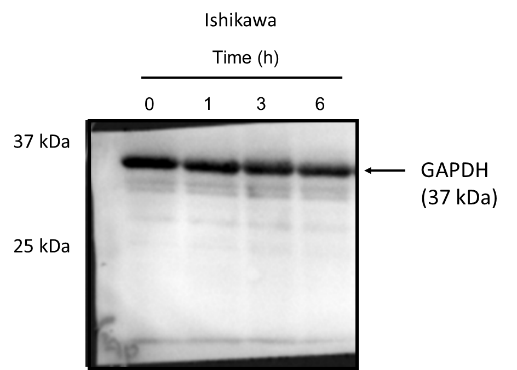

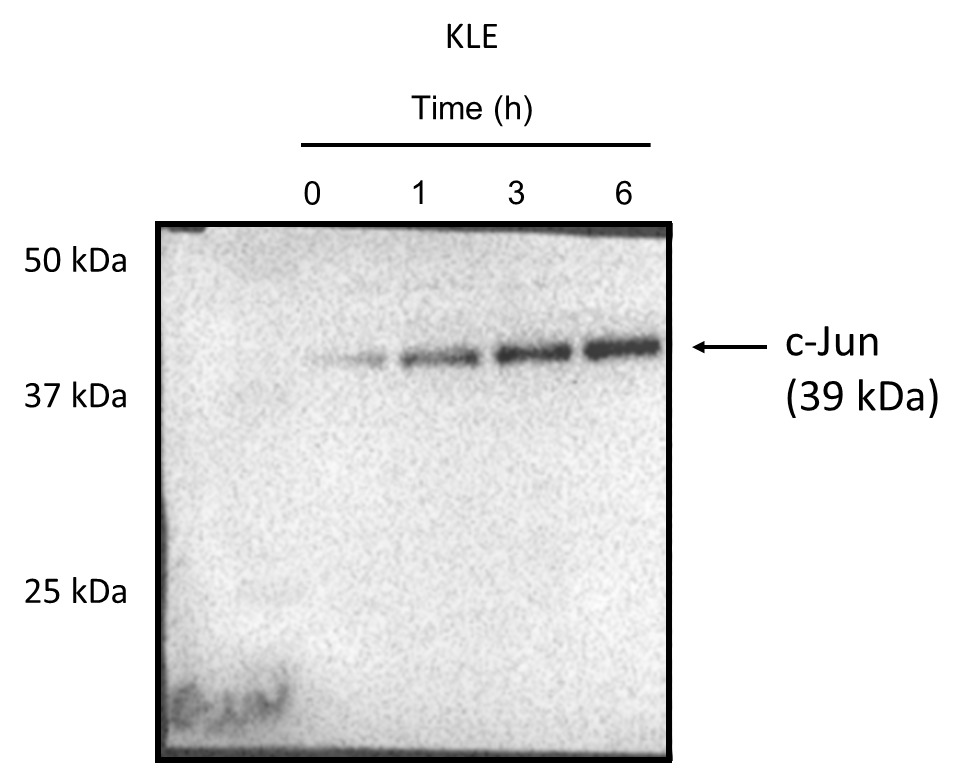
**

**
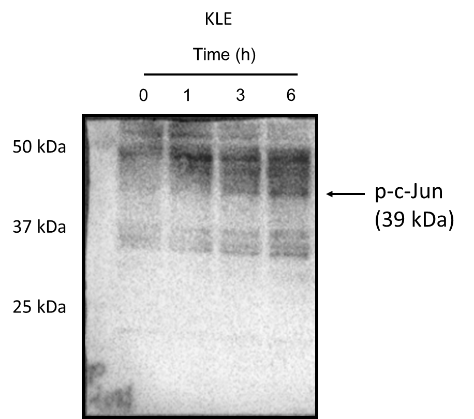

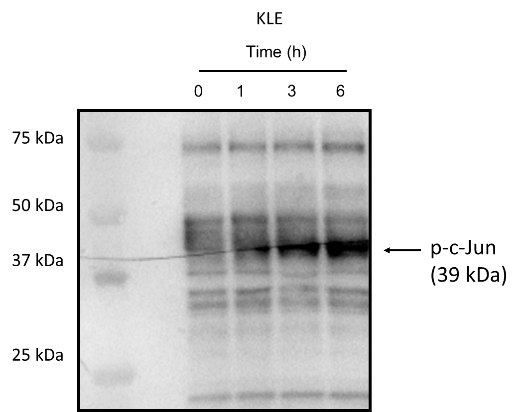
**

**
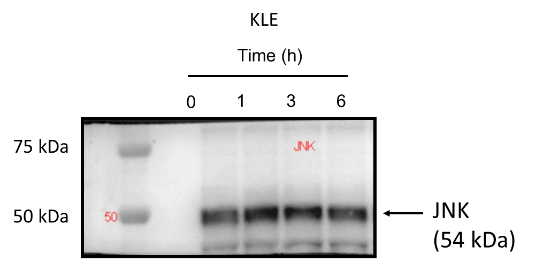

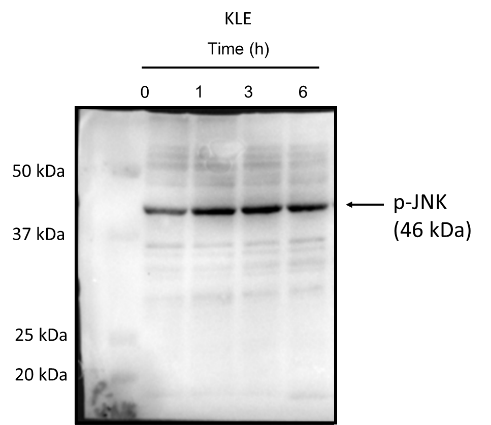

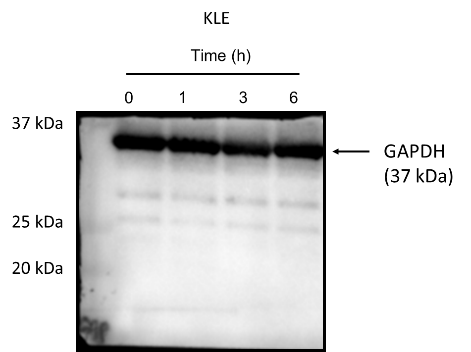
**

**Supplementary Fig. 7:** Full-length western blots of c-Jun, p-c-Jun, JNK, p-JNK, and GAPDH protein expression for Figure 4E.

**Supplementary Figure 8**

**Supplementary Fig. 8:** Apoptotic cells in the tumor sections were detected by TUNEL. The apoptotic index was defined as the percentage of immunopositive cells.

**Supplementary Table 1**

(A) Tumor volume (mm^3^) for Figure5A

(B) The ratio of tumor volume to the baseline value for Figure 5A
